# A Systematic Review and Network Meta-Analysis of Biomedical Mg Alloy and Surface Coatings in Orthopedic Application

**DOI:** 10.1155/2022/4529520

**Published:** 2022-03-31

**Authors:** XinYue Lu, HongXin Cai, Yu Ru Li, Xinru Zheng, Jiahao Yun, Wenhui Li, XiaoYu Geng, Jae-Sung Kwon, Heng Bo Jiang

**Affiliations:** ^1^The Conversationalist Club, School of Stomatology, Shandong First Medical University and Shandong Academy of Medical Sciences, Tai'an 271016, Shandong, China; ^2^Department and Research Institute of Dental Biomaterials and Bioengineering, Yonsei University College of Dentistry, Seoul 03722, Republic of Korea

## Abstract

Magnesium alloys have great application prospects as ideal bone implant materials. However, their poor corrosion resistance limits their clinical orthopedic application. Surface modification promotes the corrosion resistance of magnesium. Conversion coatings, such as calcium phosphate (Ca-P) coating, microarc oxidation (MAO) treatment, and fluoride (FLU) treatment, have been extensively investigated in in vivo studies. This systematic review and network meta-analysis compared the influence of different conversion coatings on bone repair, material properties, and systemic host response in orthopedic applications. Using the PICOS model, the inclusion criteria for biodegradable magnesium and its alloys were determined for in vivo studies. Four databases were used. The standard and weight mean differences with 95% confidence intervals were used to analyze new bone formation and degradation rate. Network structure and forest plots were created, and ranking probabilities were estimated. The risk of bias and quality of evidence were assessed using SYRCLE, CERQual, and GRADE tools. In the qualitative analysis, 43 studies were selected, and the evaluation of each outcome indicator was not entirely consistent from article to article. In the quantitative analysis, 21 articles were subjected to network meta-analysis, with 16 articles on implant degradation and 8 articles for new bone formation. Additionally, SUCRA indicated that Ca-P coating exhibited the highest corrosion resistance, followed by FLU treatment. MAO demonstrated the best capability for new bone formation, followed by Ca-P coating. Ca-P coating exhibited the highest overall performance. To conclude, coated Mg can promote better new bone formation than bare Mg and has considerable biocompatibility. Ca-P-coated Mg and MAO-coated Mg have the greatest potential to significantly promote corrosion resistance and bone regeneration, respectively. The findings of this study will provide a theoretical basis for the investigation of composite coatings and guidance for the orthopedic application of Mg bone implants.

## 1. Introduction

Magnesium alloys are biodegradable metallic bone graft materials with mechanical properties (e.g., density, elastic modulus, yield strength, and compressive strength) similar to those of natural human bones [1, 2]. Multiple studies have demonstrated that Mg alloys have excellent biocompatibility, osteoconductivity, osteoinductivity, antibacterial, anti-inflammatory, and other biological properties [3]. Owing to these excellent characteristics, Mg alloys have a wide range of application prospects in orthopedic applications and can be used for bone fracture, bone defect healing, and guided bone regeneration [2]. An Mg alloy can be safely degraded in vivo over time, after support and protection. Without the need for second operation [4, 5], it can reduce the suffering of patients, particularly the elderly who are prone to osteoporosis and children. Owing to the decrease in metal particles and the stay of ions, Mg alloys cause less toxic side effects [6–8], which reduce the risks of the stress shielding effect [9] as well as the possibility of a second fracture after surgeries [10, 11]. Furthermore, Mg alloys have considerable potential as internal fixation materials for fracture [12]. In addition, the Mg ions produced after degradation are common in vivo metabolites, which are stored in the bones; therefore, within the normal range [13], magnesium ions are not toxic to the human body.

Despite various advantages, poor corrosion resistance limits the extensive application of Mg alloys. Therefore, current research on magnesium has focused on improving their corrosion resistance. There are two main ways to improve corrosion resistance, i.e., alloying [14] and coating [15].

Alloying can optimize the composition and performance of Mg alloys and is an effective but costly strategy [16–18]. It may also cause deterioration under certain specific conditions. Compared to alloying, coating is an economical and effective strategy. Surface modification forms a corrosion-resistant film on the surface of the Mg alloy substrate and prevents the external medium from corroding the Mg alloy, which results in improved corrosion resistance [18]. To date, various coatings have been developed, and surface modifications can be divided into two types according to the formation mechanism: conversion coatings and deposition coatings [19].

Conversion coating is an in situ coating formed by the chemical reaction between the Mg alloy substrate and the solution, which involves the complex interaction of metal dissolution and precipitation. Chemical or electronic coating techniques involving salts and oxides result in a strong adhesion between the magnesium substrate and coatings, which reduces the risk of the surface coatings peeling off [20, 21].

Multiple clinical studies have confirmed that animal experiments are essential to evaluate the safety and properties of biomaterials and relevant medical equipment [22]. It is difficult for in vitro simulations to evaluate in vivo performances because the alloying elements and coating materials affect the mechanical and corrosion properties, coatings, and experimental environments, such as different buffer systems and ions [13]. Compared to in vitro studies, animal experiments provide a closer body fluid environment to real humans; therefore, they can predict the information transformation between the experimental results and human clinical environment. Animal experiments are essential for evaluating bone repair, material properties, and host responses in the system.

Despite the wide range of clinical application prospects, evidence-based medicine seldom focuses on the surface modification of Mg alloys. Therefore, animal experiments involving widely applied conversion coatings were selected to perform a systematic review and network meta-analysis of chemical conversion coatings, such as calcium phosphate (Ca-P) conversion coating, fluoride (FLU) conversion coatings, and anodic oxidation, also known as microarc oxidation (MAO) coatings [19, 21]. Unlike a traditional literature review, this systematic review aims to perform qualitative and quantitative analyses based on relevant research [23], has strict inclusion criteria, and requires standardized instrument [24]. As far as we know, there has not been any systematic review on Mg coatings and no meta-analysis, quantitative evaluation, has been done yet. A network meta-analysis was performed to screen for magnesium alloy surface modification technologies with better comprehensive performance. The quality of the included literature was evaluated, and the evidence was graded based on the research results. Our research explored and compared the effects of magnesium alloy coating in preclinical trials and evaluated the feasibility of clinical transformation and follow-up clinical trials. The aim of this systematic review is to evaluate the surface coatings for biomedical Mg in orthopedic application with quantitative assessment.

## 2. Materials and Methods

### 2.1. PICOS

Patients: animal models with bone defect treatment, no restriction on animal species and surgical sites.

Intervention: Mg alloys with surface conversion coatings, including Ca-P conversion coating, FLU conversion coating, and MAO coating.

Comparator: Mg alloys without surface coating treatment, bare Mg (BM).

**Figure 1:**
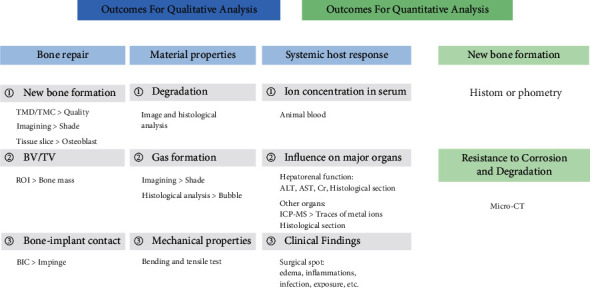
Outcome assessment.

Outcomes: qualitative and quantitative analyses of the outcome indicators are shown in Figure 1.

Study design (randomized controlled trials): the specific inclusion and exclusion criteria are given in Table 1.

### 2.2. Literature Screening

**Figure 2:**
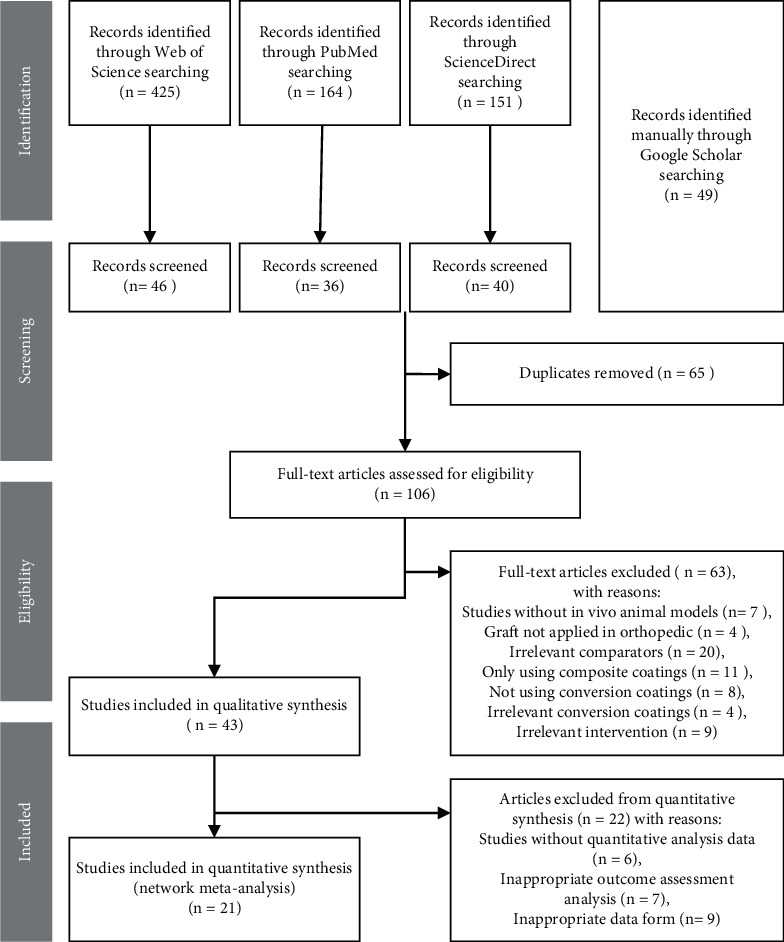
PRISMA flowchart.

Studies that fit these criteria were retrieved during this process. Searches were conducted on PubMed, Web of Science, and ScienceDirect databases, and a manual search was performed using Google Scholar.  Table 2 provides the search strategies used in the Web of Science, and the searching strategies in other databases are given in Tables S1–S3 in Supplementary Materials. We performed the first screening in Endnote (X 9.3.1). First, the titles and abstracts were read, and duplicate studies were removed. Next, the full texts were read, and a second screening was performed based on the inclusion and exclusion criteria. The processes are shown in the PRISMA flowchart (Figure 2). Each step of the retrieval process and the results are presented in table. Literature screening was performed independently by the two authors, and divergences were resolved through discussion or consultation with another author.

### 2.3. Data Extraction

The general characteristics of the included studies were collected independently by two authors and then recorded on a predesigned list. Extracted data included species, ages, and weights of the animal models, types of bone defects (including fracture, osteotomy, and guided bone regeneration), types and shapes of graft materials, Mg alloy components, and the first authors of the original documents. A third author compared the consistency of the data.

### 2.4. Risk of Bias of Individual Studies

To assess the effectiveness of interventions in animal studies and the methodological quality of the studies, the original studies were evaluated using the Systematic Review Center for Laboratory Animal Experimentation (SYRCLE) assessment tool [25], which is a tool for evaluating the quality of animal studies based on the development of the Cochrane risk bias assessment tool [26]. We evaluated each of the 10 items using Review Manager 5.4 software. The processes were independently conducted by the two authors, and any differences were resolved through discussion or consultation with another author.

### 2.5. Quality of the Evidence and Risk of Bias across Studies

Given the differing nature of qualitative and quantitative evidence, we used confidence in the evidence from reviews of qualitative research (CERQual) and Grading of Recommendations Assessment, Development, and Evaluation (GRADE), respectively, to grade and evaluate the evidence obtained in this study [27–29]. The CERQual tool aims to objectively evaluate and describe the credibility of qualitative evidence review results in four aspects: method limitations, relevance, consistency, and data adequacy [27]. For network meta-analysis, based on the principal principles of GRADE, the risk of bias was integrated with the included data [30], and the GRADE tool was used to evaluate the research limitations, indirectness, inconsistency, inaccuracy, and publication bias of the evidence obtained from different outcome indicators. To assess publication bias, we created a funnel diagram for visual observations.

### 2.6. Data Synthesis and Network Meta-Analysis

In the network meta-analysis, we evaluated two outcome indicators: new bone formation and degradation rate (% degradation). As a continuous data variable, the standardized mean difference (SMD) was used to assess new bone formation. Two main indicators measured new bone formation: new bone volume and new bone formation rate. Although the units were not uniform, they had the same concept at different scales, so we calculated the SMDs of the included studies to evaluate new bone formation. The SMD was calculated using an appropriate 95% confidence interval (CI) for consolidation analysis. For % degradation, weighted mean differences (WMDs) were used for the statistical analysis of % degradation assessment. WMDs were calculated with appropriate 95% CIs for the synthesis analysis to evaluate % degradation.

To visualize the connection between different Mg alloy surface chemical treatment coating technologies, network evidence maps were established to determine whether there is a dominant sample study and visualize the network structure [31].

Traditional pairwise meta-analysis of all direct comparisons was conducted using a random effect model. The network estimations, which aggregated direct and indirect data estimation for each intervention, are shown in the forest plots [32]. Visual inspection of forest plots and *I*^2^ measurements were used to infer the heterogeneity [33]. The mean rank and surface under the cumulative rank area (SUCRA) of different surface treatment techniques for Mg alloy were calculated [34], estimating the ranking probability of all surface coatings at each possible rank. A comprehensive comparison was conducted after mixing the results of each outcome indicator to observe the mixed effect of different coatings in vivo.

## 3. Results

### 3.1. Study Selection

Four databases were used. Three databases were searched with established search strategies, including Web of Science, PubMed, and Science Direct. We also manually searched Google Scholar based on the research purpose and the PICOS model. The specific procedure is shown in Figure 2. After filtering the title and abstract and removing the repeated literature, the full text of 106 articles was read. The included and excluded studies after first screening are given in Table S4 in Supplementary Materials. Forty-three articles were included in the systematic review, and twenty articles were included in the quantitative network meta-analysis.

### 3.2. Characteristics of the Included Studies

**Figure 3:**
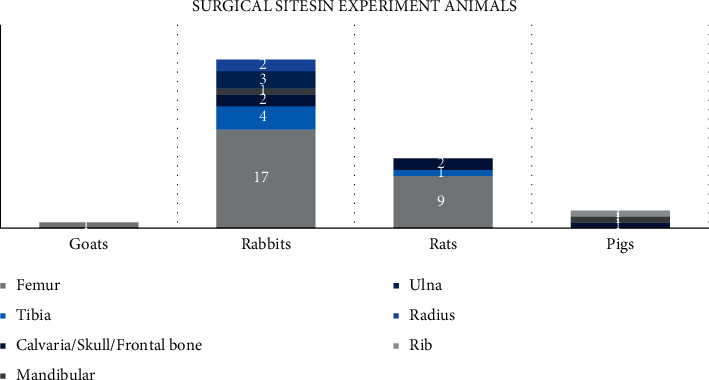
Animal models and surgical sites in included animals.

Four types of animals were included in our study: rabbits, rats, goats, and pigs; seven surgical sites were included: femur, tibia, calvaria, mandibular, ulna, radius, and ribs (Figure 3). Among all the surgical sites, the tibia is the most frequent, and among all the animals, rabbits are the most chosen.

Table 3 provides detailed information about the included studies: Mg alloy type, shape, and size, and the animal models used in the studies.

In the qualitative analysis, three directions of outcome indicators were evaluated. However, the time and assessment measurements of each outcome indicator were not identical. Forty articles concluded the influence of the materials on bone repair, forty-two articles reported the material properties after implantation, and thirty-two revealed the system response of the host.

In the quantitative analysis, twenty-one articles were included in the network meta-analysis. Among them, a total of eight articles were based on new bone formation, and sixteen articles revealed the degradation situation.

### 3.3. Risk of Bias within the Studies

**Figure 4:**
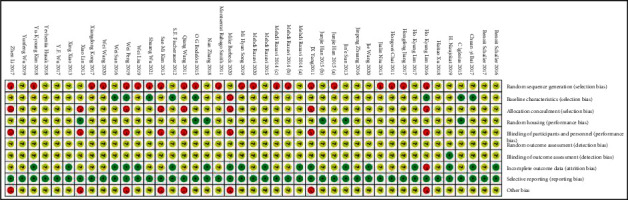
Risk of bias in each included 43 studies.

**Figure 5:**
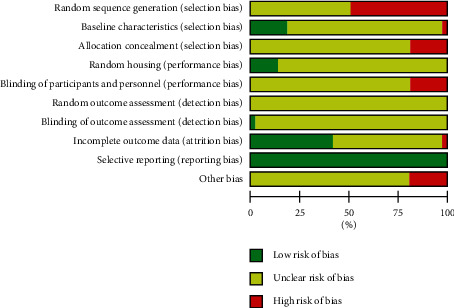
Risk of bias assessment within the 43 included studies.

In terms of the quality of the included studies, Figures 4 and 5 show the risk of bias within the studies. Of all the entries, random sequence generation was the biggest factor causing bias. Notably, 48.8% of all the included studies did not mention whether randomized distribution sequence methods were used, thereby generating a high risk of bias, since there is no description about the specific random grouping methods, and it is not possible to determine whether the generation methods and applications were correct. Furthermore, 18.6% of the studies showed a high risk of bias in distribution, research blinding, and other biases. This is mainly because a participant's body part intervened in the experiment, resulting in a unit of analysis errors. The expected outcome indicators were clearly reported in all studies, and there were no selectively reported studies.

Generally, the included studies were of low quality, and incomplete experimental information resulted in high ambiguity when assessing.

### 3.4. Synthesis Result of Qualitative Analysis

**Figure 6:**
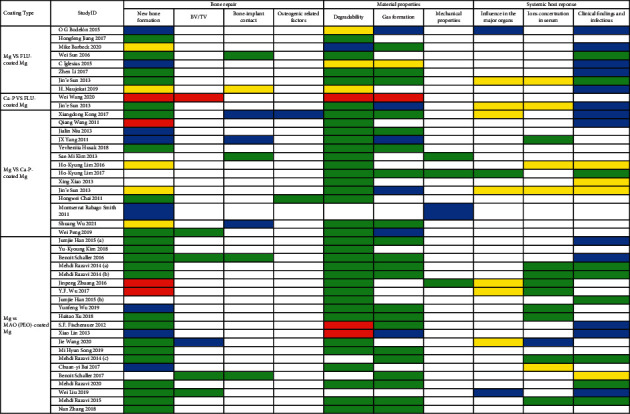
Results of the qualitative analysis. (red, control group > experimental group; green, experimental group > control group; yellow, there is no significant difference between the two groups; blue, there is no comparison between the two groups or the result of one group was solely reported).

The parameters used when manufacturing the MAO coatings are given in Table 4.  Table 5 provides the manufacturing parameters of the FLU and Ca-P coatings in the included studies. The qualitative results are shown in Figure 6.

#### 3.4.1. Orthopedic Application of FLU-Coated Mg against Bare Mg

(1) Bone repair
  (i) New bone formation
      New bone formation was described in eight studies [35–42]. Four studies indicated that FLU-coated Mg showed better new bone formation than BM [35–37, 39]. The BM had a wider range of low-density bone areas, while the FLU-coated Mg had smaller holes around it and higher bone density [36]. The tissue mineral density (TMD) and tissue mineral content (TMC) of screws in the FLU-coated Mg were better than those in BM, indicating that FLU coating had better osteoinductivity [35]. The newly formed bone trabeculae were more compact in FLU-coated Mg than in BM [37, 39]. The minor differences between the FLU-coated Mg and BM groups in two studies were not statistically significant [40, 41], and there was no indication that the FLU-coated Mg group was superior to the BM group in bone healing. New bone formation was observed in both the FLU-coated Mg and BM groups, but no comparison was made [42]. In addition, the study only described new bone formation in BM and did not elaborate on the results in FLU-coated Mg.
  (ii) Bone-implant contact
      Two studies described the bone-implant contact [35, 41]. The study demonstrated that implants have more continuous and closer contact at three months [35]. No statistically significant difference was observed between the two groups [41].
(2) Material properties
  (i) Degradation
      Eight studies described the degradation of Mg alloys [35–42]. Four studies showed that the degradation performance of FLU-coated Mg was better than that of BM [35–37, 39]. In the BM group, there was obvious corrosion and fracture, whereas the FLU-coated Mg showed significantly slower degradation, and the shape remained more intact, which could provide sufficient support for the bone [37]. No statistically significant difference in reabsorption was observed between the two groups [38, 41, 42]. The study indicated that implant residues could be detected in both groups, but no comparison was made [40].
  (ii) Gas formation
      Five studies described gas formation [37–40, 42]. Less hydrogen was observed in three studies, demonstrated by the appearance of no obvious gas hole [40], no sign of gas shadow [39], and less gas release [37]. However, in another study, gas formation and absorption were described solely in case of the BM group, and the results for the FLU-coated Mg group were not reported [38]. A reduction in gas volume was observed in both groups; however, no comparison was discussed [42].
(3) Systemic host responses
  (i) Influence on the major organs
      Two studies described the influence of grafts on major organs [39, 42]. The results of the FLU-coated Mg group were not specified in this study [42]. The study showed that the physiological functions of the kidney and liver were not affected by FLU-coated Mg and BM at three months, and no statistically significant difference was observed between the two groups [39].
  (ii) Ion concentration in serum
      Only one study described the ion concentration in serum [39]. The study showed that the degradation of both groups did not affect the Ca^2+^ levels in serum, and no statistically significant difference was observed for the increase in serum Mg^2+^ [39].
  (iii) Clinical findings
      Two studies described superior infection resistance in the FLU-coated Mg group compared to the BM group [35, 39]. In the BM group, inflammatory cell infiltration was observed at both one and two weeks; local bleeding was also observed. In the FLU-coated Mg group, there was only local inflammatory cell infiltration at one week, no inflammatory response and bleeding symptoms until two months, and no inflammatory response was observed at three months. The study revealed no obvious sign of inflammation in either group at three months. However, more Mg particles were swallowed by phagocytes in the BM group during the degradation process, and only a few small Mg particles were observed in the FLU-coated Mg group [39]. Five studies showed no foreign cells or signs of inflammation in the FLU-coated Mg or BM group. However, no comparison has been made in other studies [37, 38, 40–42].

#### 3.4.2. Orthopedic Application of FLU-Coated Mg against Ca-P-Coated Mg

(1) Bone repair
  (i) New bone formation
      Two studies described new bone formation [39, 43]. One study showed that the FLU-coated Mg group exhibited better new bone formation and more compact arrangement of bone trabeculae than the Ca-P-coated Mg group at three months [39]. The trabecular number (TB. N) of the FLU-coated Mg group was significantly lower than that of the Ca-P-coated Mg group at four and eight weeks, which shows poorer bone regeneration ability of the FLU-coated Mg group than the Ca-P-coated Mg group [43].
  (ii) BV/TV
      Only one study described BV/TV [43]. The BV/TV in the FLU-coated Mg group was significantly lower than that in the Ca-P-coated Mg group at four and eight weeks [43].
(2) Material properties
  (i) Degradation
      Two studies described bone implant degradation [39, 43]. One study showed that the FLU-coated Mg group degraded faster and produced a larger amount of H_2_ compared with the Ca-P-coated Mg group at four and eight weeks [43]. The opposite results were obtained in another study, and the degradation rate of the FLU-coated Mg group was significantly lower than that of the Ca-P-coated Mg group at three months [39].
  (ii) Gas formation
      Two studies described gas formation [39, 43]. One study showed that a large amount of H_2_ appeared in the degradation process of FLU-coated Mg at both four and eight weeks, but not in the Ca-P-coated Mg group [43]. Another study revealed that gas shadow was not observed around Mg alloy grafts in both groups, but no comparison was made [39].
(3) Systemic host responses
  (i) Influence on the major organs
      Only one study described the influence of Mg alloy bone grafts on major organs [39]. Neither the FLU-coated Mg nor the Ca-P-coated Mg impacted the physiological functions of the kidney and liver at three months, and no statistically significant difference was found between the two groups.
  (ii) Ion concentration in serum
      Only one study reported ion concentrations in serum [39]. The degradation of neither the FLU-coated Mg nor the Ca-P-coated Mg affected serum Ca^2+^, and no statistically significant difference was observed for the increase in serum Mg^2+^ [39].
  (iii) Clinical findings
      Only one study reported clinical findings. No significant signs of inflammation were observed in the FLU-coated Mg and Ca-P-coated Mg groups at three months, while some small magnesium particles were observed. In addition, no comparison was made [39].

#### 3.4.3. Orthopedic Application of Ca-P-Coated Mg against Bare Mg

(1) Bone repair effects
  (i) New bone formation
      Eleven studies described new bone formation [39, 44–53]. Four studies revealed better new bone formation in the Ca-P-coated Mg group [44, 48–50, 53]. Histological staining showed better osteoinductivity [44]. The adhesion, proliferation, and differentiation of bone cells in the Ca-P-coated Mg group were significantly enhanced [48]. The *β*-TCP coating has great biocompatibility and osteoconductivity on the biological surface, and more bone matrix and interconnected trabecular bone in the newly formed tissue were observed [50]. However, another study revealed opposite results: compared with the BM group, less newly formed tissue was discovered around Ca-P-coated Mg [45]. Three studies revealed that there is no sign that the Ca-P-coated Mg was better than BM in new bone formation, since there was no statistically significant difference between these two groups. Wu et al. postulated that this may have resulted from the decrease in the Ca-P coating over time [52]. Moreover, three studies revealed that new bone formation can be seen in both groups [46, 47, 51], but no comparison has been made between the two groups. The radiological healing score was used to assess the recovery conditions, and the animals in the Ca-P-coated Mg group showed better overall recovery [51].
  (ii) BV/TV
      Only one study described BV/TV [53]. During the experimental period, BV/TV of all groups increased, and Ca-P-coated Mg showed the highest BV/TV value, while BM performed worse [53].
  (iii) Bone-implant contact
      Four studies showed bone-implant contact [44, 47, 52, 54]. Only one study revealed the bone-implant contact around Ca-P-coated Mg.
      Coated Mg was better than that around BM. In the middle stage of the implantation, the contact between the bone tissue and implant in the coated Mg group was significantly higher than that in the BM group, while the Ca-P-coated Mg group was slightly higher in the later stage [54]. Three studies described the interface between bone tissue and implants without comparing the two groups [44, 47, 52]. In the 18 months after the operation, close contact was observed around the Ca-P-coated Mg, which indicates excellent osseointegration [44]. The formation of new bone was in close contact with the Ca-P-coated Mg, which demonstrates that the higher compatibility of the Ca-P coating to osteoblasts can lead to tight osseointegration at the implant-host tissue interface [55].
(2) Material properties
  (i) Degradation
      Thirteen studies described implant degradation [39, 44–50, 52–54, 56, 57]. All studies reported that the corrosion resistance of Ca-P-coated Mg was better than that of BM. Severe corrosion was observed in the BM group, while the Ca-P-coated Mg group maintained a clear shape [39, 44–47, 50, 53, 54]. In the later stage, the hydroxyapatite-coated Mg had a relatively active degradation, indicating that in the early stage, Ca-P-coating has significant advantages in resisting absorption [49]. The residual volume of the Ca-P-coated Mg was significantly higher than that of BM [49, 52], and the average mass loss was significantly smaller than that of BM [57], indicating that Ca-P coating has a better protective effect.
  (ii) Gas formation
      Eight studies described gas formation [39, 44, 46–48, 52, 53, 56]. Less hydrogen was observed in the Ca-P-coated Mg group in five studies [44, 46, 48, 52, 56], indicated by no gas shadow [44], no obvious gas [46, 56], no voids or local accumulations formed by hydrogen [48], and smaller cavity [52]. In the remaining three studies, two revealed no bubbles [47] or gas shadow [39] in either group, and one revealed visible subcutaneous airbags on the surface of the membrane [53].
  (iii) Mechanical properties
      Three studies described the mechanical properties of the graft [51, 54, 56]. Two studies indicated that Ca-P-coated Mg exhibited better mechanical properties than BM [54, 56]. After implantation of four weeks, the tensile strength of BM was approximately half of the initial value, while Ca-P-coated Mg had a slightly smaller decrease after 12 weeks [54]. Ca-P-coated Mg can still maintain a tensile strength of >190 MPa, while the tensile strength of BM decreases rapidly after two weeks [56]. Although there is no comparison between Ca-P-coated Mg group and BM group, the treated ulna possessed biomechanical properties similar to those under normal conditions, revealing that the coated Mg alloy performed well in bone fracture surgery [51].
(3) Systemic host responses
  (i) Ion concentration in serum
      Three studies described ion concentrations in serum [39, 47, 49]. The serum Mg^2+^ level in the Ca-P-coated Mg group was lower than that in the BM group [47]. Two studies showed that there were no statistically significant differences in the changes in ion concentration [39, 49]. Serum Mg^2+^ levels were in the normal range at various time periods [49]. The degradation of coated magnesium had no negative effect on the increase in serum Mg^2+^ and Ca^2+^ [39].
  (ii) Influence on the major organs
      Three studies described the influence of Ca-P-coated Mg in major organs [39, 44, 56]. It has been reported that Ca-P-coated Mg groups have less impact on major organs [56]. Two studies reported that no statistically significant difference was observed in both groups [39, 44]. The graft in both groups had no side effects on the liver and kidney functions of goats [44]. According to the hematological analysis of rabbit serum, it was found that the changes in ALT and urea in Ca-P-coated Mg and BM groups were not statistically significant. Visceral section observation revealed that the liver and kidney functions were normal, demonstrating that Ca-P coating has great in vivo biocompatibility [39].
  (iii) Clinical findings
      Seven studies described clinical findings [39, 44, 45, 49, 50, 56, 57]. Two studies revealed that Ca-P-coated Mg groups have better antiinfection ability [50, 56]. Compared with the BM group, there was no plate exposure during the experiment in the Ca-P group. Three studies revealed no infection or inflammation, and there was no significant difference between the two groups [44, 45, 49, 57]. The average animal weight in the Ca-P-coated Mg group increased, while that in the BM group decreased [57].

#### 3.4.4. Orthopedic Application of MAO-Coated Mg Compared to Bare Mg

(1) Bone repair
  (i) New bone formation
      Twenty studies described new bone formation [11, 55, 58–75]. Fifteen studies reported that the MAO-coated Mg group was superior to the BM group [11, 55, 58–62, 65, 67, 70, 71, 73–75]. In the MAO-coated Mg group, the cortical bone healed well, and a complete fracture callus was formed while the fracture space was connected closely [58]. Histological analysis showed that the MAO-coated Mg group had more new bones [55, 59–61, 70, 71, 73–75], higher bone layer density [67], and a more perfect overall structure [62]. Osteoblasts and new bone formation were observed in the corrosion pit in the MAO-coated Mg group, whereas only stroma and fibrous tissue of fibroblasts were observed in the BM group [68]. BM significantly interfered with the growth of the physical structure of the distal femur, but not in the MAO-coated Mg group [71]. In contrast, two studies showed that new bone formation in the BM group was better than that in the MAO-coated Mg group [63, 64]. Histological examination showed that the BM had a higher bone needle maturity and better bone healing [63]. Histological analysis showed that the maturity and continuity of the trabecular bone in the BM group were higher than those in the MAO-coated Mg group, and the callus growth and fracture healing rate were also better [64]. Three studies reported bone formation in each group, but there was no comparison between the two groups [66, 69, 72].
  (ii) BV/TV
      Four studies described BV/TV [60, 70, 73, 76]. The bone mineral density around the MAO-coated Mg was much higher than that around BM [60, 73, 76]. No significant differences were found between the two groups [70].
  (iii) Bone-implant contact
      Two studies described the bone-implant contact [60, 76]. Both these studies showed that the bone contact area of implants was higher in the MAO-coated Mg group [63].
(2) Material properties
  (i) Degradation
      Eighteen studies described implant degradation [11, 55, 58–70, 72, 74, 75]. Sixteen studies reported that the degradation performance of MAO-coated Mg was better than that of BM [11, 55, 58–67, 70, 72, 74, 75]. Severe corrosion occurred in the BM group, while only slight degradation occurred in the MAO-coated Mg group, and the degradation rate was significantly lower [11, 58, 63–67, 75]. By measuring the residual volume [59, 60, 70, 72] or weight loss [55, 61, 62, 74], it was found that the MAO-coated Mg group exhibited less degradation and improved corrosion resistance. In contrast, two studies reported that the degradation performance of the BM group was better than that of the MAO-coated Mg group [68, 69]. In the middle stage of the experiment, the degradation rate of the MAO-coated Mg group accelerated, resulting in the final dissolution of the implants in the MAO-coated Mg group earlier than that in the BM group [68]. In the early stage of the experiment, the degradation rate of the MAO-coated Mg group was faster than that of the BM group, whereas in the later stage, the degradation rates in both groups were accelerated. Overall, the corrosion resistance of MAO-coated Mg was better than that of BM [69].
  (ii) Gas formation
      Fourteen studies described gas formation [11, 55, 58, 60–62, 66–69, 71, 74–76]. Thirteen studies reported less gas generation in the MAO-coated Mg group than in the BM group [11, 55, 58, 60–62, 66–68, 71, 74–76]. During the experimental period, fewer bubbles were formed in the MAO-coated Mg group [61, 62, 66] and there was no obvious gas [58, 67], or the generated gas was less than that of the BM group [11, 55, 60, 69, 71, 74–76]. In addition, only one study reported the gas volumes detected at different times in the two groups, but no overall comparison was made [68].
  (iii) Mechanical properties
      Only one study conducted a three-point bending test of implants [63]. The bending strength and tensile strength of the MAO-coated Mg were significantly higher than those of BM at each follow-up time, and only the MAO-coated Mg with a thicker coating reached or even exceeded the mechanical strength of the human femur.
(3) Systemic host responses
  (i) Influence on the major organs
      Four studies described the influence of Mg alloy bone implants on major organs [63, 64, 70, 73]. Three studies reported no statistical difference between the two groups [63, 64, 70]. Histological evaluation or pathological examination showed that there were no abnormal changes in the volume and shape of glomeruli, the shape of renal tubules, the arrangement of myocardial fibers, and the volume and shape of cardiomyocytes; there was no cell proliferation or necrosis. No obvious abnormalities were found in the heart and kidney tissues, indicating that there were no adverse reactions in the circulatory and urinary system [63, 64], but the structure of the hepatic sinus was damaged to a certain extent and renal interstitial hemorrhage [70]. One study reported that there is no evidence that the implants used were toxic to major organs, and no pathological changes were observed; however, no comparison was made between the two groups [73].
  (ii) Ion concentration in serum
      Ten studies described ion concentrations in serum [61–64, 66, 67, 70–72, 74]. Eight studies reported that MAO-coated Mg has a lower serum magnesium concentration than BM [61–64, 66, 67, 71, 74]. The Mg^2+^ concentration in the two groups increased with the culture time, but Mg^2+^ concentration in the BM group increased significantly more than that in the MAO-coated Mg group. The difference in serum magnesium values before and after surgery was less than that in the BM group [61–64, 66, 67, 71, 74]. One study reported that there was no significant difference in serum Mg^2+^ concentrations between the two groups [72]. Another study reported that implants did not cause metabolic disorders of major ions, such as sodium, magnesium, and calcium [70], but there was no comparison between the two groups.
  (iii) Clinical findings
       Twelve studies described clinical findings [55, 58, 60–62, 65, 68, 71, 73, 74, 76]. Six studies reported that MAO-coated Mg exhibited better antiinfectious properties than BM [55, 61, 62, 65, 71, 74]. More severe inflammatory reactions were observed in the BM group than in the MAO-coated Mg group [55, 61, 71, 74]. The high corrosion of Mg alloys produces a large amount of hydrogen and inhibits the physiological bone reaction [62]. In the BM group, rapid degradation causes the formation of absorption lacunae in bone tissue, which produces inflammation, activates osteoclasts and inhibits osteoblasts, induces osteolysis, and causes allergic reactions [65]. One study reported that there was no inflammatory reaction in each group [76]. A slight inflammatory reaction occurs in bone tissue with MAO-coated Mg, and the backbone condition of BM has not been reported [69]. No foreign body cells or signs of inflammation were observed in the two groups [58, 60, 68], and no animal death or infections occurred [73], but there were no comparisons between the two groups.

### 3.5. Network Structure and Results Synthesis in Network Meta-Analysis

**Figure 7:**
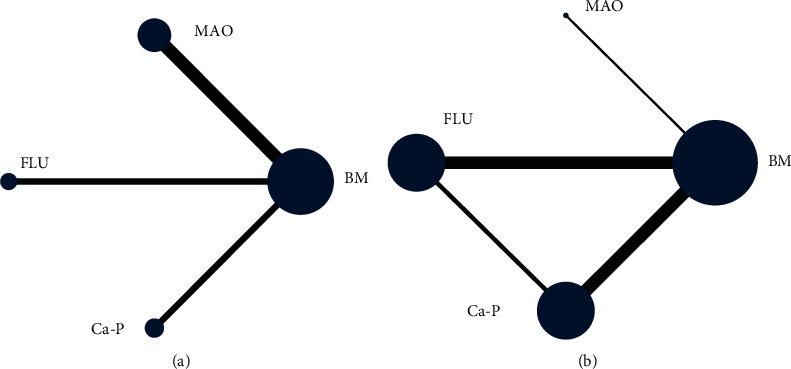
Network structure (a) % degradation and (b) new bone.

In order to investigate the effects of different coating technologies in orthopedic applications, we conducted the experiments at different follow-up times in each original research to launch meta-analysis; this is shown in the network structure (Figure 7), which depicts the network structure of new bone formation and % degradation outcome assessment. Each dot represents an intervention, and the line between represents a direct comparison between the two different interventions. The size of the dots is proportional to the number of direct comparisons. Four interventions were included in the figure. In the degradation outcome assessment of the comparisons between each intervention, MAO is the most often used surface modification technology, while studies involving FLU treatment are limited. In the assessment of new bone outcomes, FLU treatment is the most frequently assessed coating technology. As for research on MAO coating, they seldom focused on new bone formation.

**Figure 8:**
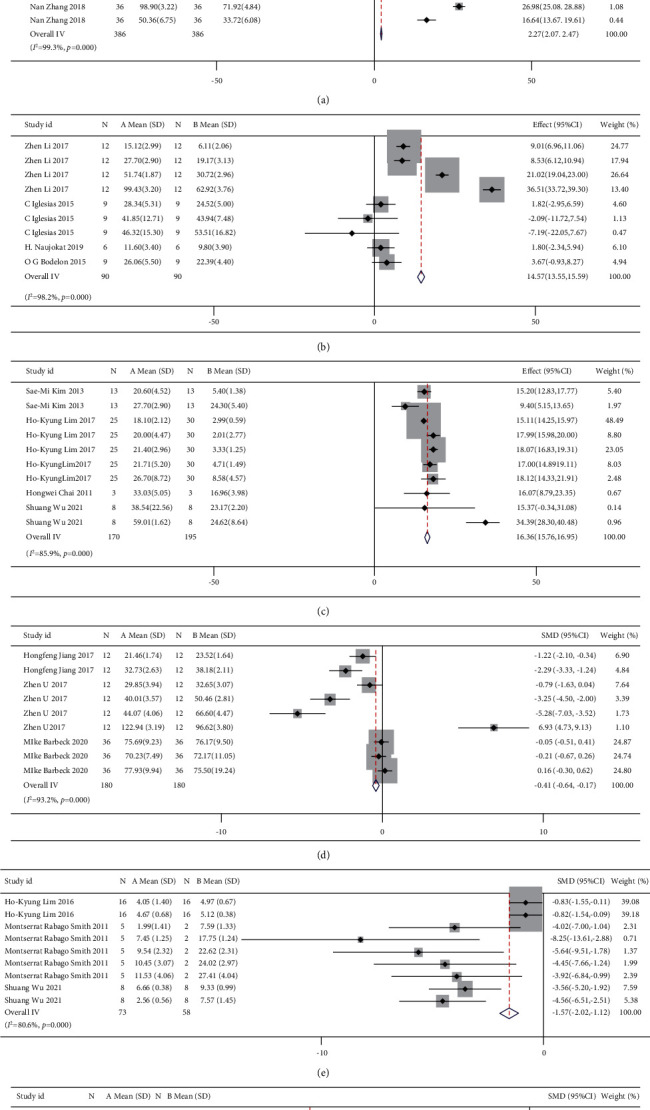
Forest plots of direct comparisons: (a) % degradation for BM vs. MAO-coated Mg, (b) % degradation for BM vs. FLU-coated Mg, (c) % degradation for BM vs. Ca-P-coated Mg, (d) new bone formation for BM vs. FLU-coated Mg, (e) new bone formation for BM vs. Ca-P-coated Mg, and (f) new bone formation for FLU-coated Mg vs. Ca-P-coated Mg.

**Figure 9:**
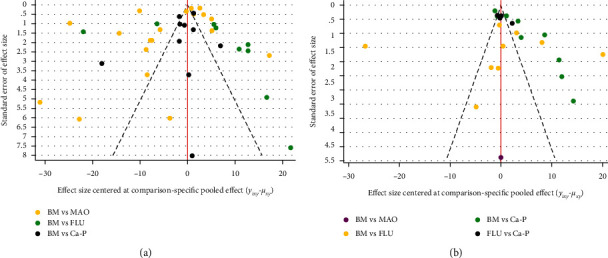
Funnel plot to investigate heterogeneity and publication bias: (a) % degradation and (b) new bone.

Traditional pairwise meta-analyses were conducted, and the results are shown in Figure 8 for the direct comparison in % degradation and new bone formation outcome assessment through forest plots. The three coatings exhibited excellent properties compared to BM in terms of the new bone and % degradation. However, the heterogeneity was large, indicating that the results obtained were not reliable. In addition, for both outcome assessments, the dots beyond the funnel plot indicate heterogeneity between the original studies (Figure 9).

**Figure 10:**
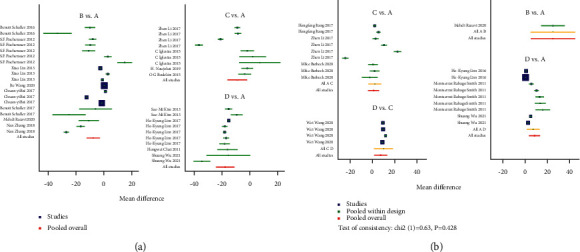
Forest plots for all comparisons: (a) % degradation and (b) new bone formation. A, BM; B, MAO-coated Mg; C, FLU-coated Mg; D, Ca-P-coated Mg.

**Figure 11:**
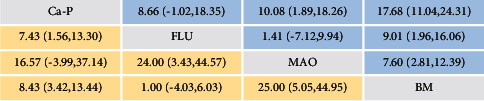
Mixture estimate confidence interval of different outcome assessments; yellow on the left represents the outcome of new bone, and the blue on the right represents the % degradation assessment.

**Figure 12:**
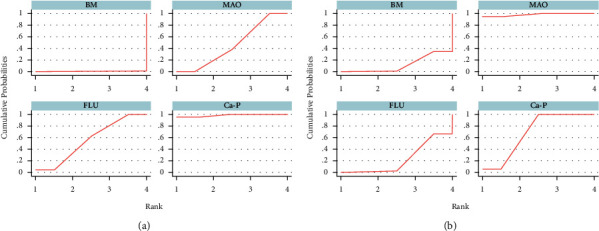
Plot of surface under cumulative ranking curves for all treatments: (a) % degradation and (b) new bone.

**Figure 13:**
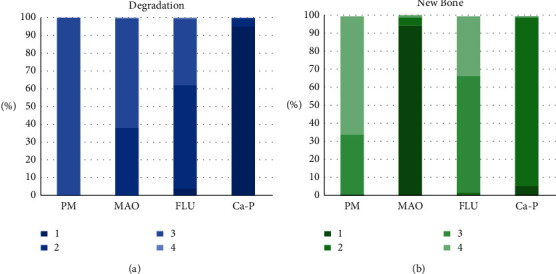
Ranking probabilities of different treatment under each outcome assessment: (a) % degradation and (b) new bone.

**Figure 14:**
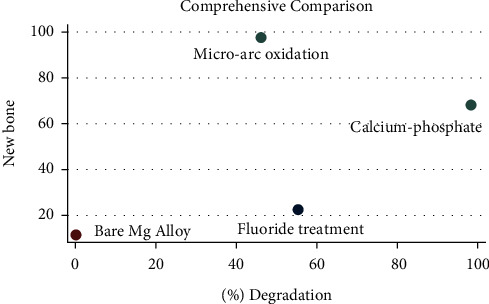
Comprehensive comparison of different interventions.

A network meta-analysis was also performed. Figure 7 shows the interval mixture comparison between each intervention for different outcomes. Because there were no closed loops in the degradation assessment network, the consistency of the network could not be tested. The outcome assessment for new bone follows the consistency of *P*=0.428 and *X*^2^ (1) = 0.63. The overall forest plots in both networks and test of inconsistency is shown in Figure 10.  Figure 11 has shown the mixture estimate confidence interval of different outcome indicators. In Figure 12, the SUCRA plots showed that Ca-P coating exhibited the best degradation, followed by FLU treatment. In terms of the new bone, MAO showed the best results, while the second was Ca-P coating. The treatment ranking possibility showed the same results (Figure 13). The darker color represents better performance in the corresponding outcome. A comprehensive comparison of the four interventions shows that the Ca-P coating has the best comprehensive effect (Figure 14). The contribution plots of each direct comparison are shown in Figures S1 and S2 in Supplementary Materials.

### 3.6. Risk of Bias across Studies and Confidence of the Evidence

The funnel plots in Figure 9 show publication bias, which demonstrates that in terms of % degradation, other original studies have demonstrated obvious publication bias, except for the comparison involving Ca-P coating. However, in the outcome assessment of new bone formation, except for the comparison involving Ca-P coating, there was no publication bias in other original studies. The quality of the qualitative evidence is given in Table 6, and the quality of the quantitative evidence is given in Table 7.

## 4. Discussion

### 4.1. Orthopedic Application of MAO-Coated Mg Alloy

MAO coating, also known as plasma electrolytic oxidation (PEO), is a high-pressure plasma-assisted anodic oxidation process widely used in surface modification of Mg alloy [77–92]. The MAO coating process produces a unique porous structure on the surface that slows down the corrosion rate of Mg alloys, increasing the adhesion strength between implants and cells [21], and the adhesion of organic or polymer surface coatings [92]. Previous studies have demonstrated that MAO coatings have the advantages of high hardness, excellent wear resistance, moderate corrosion, good thermal stability, and dielectric properties [77, 86, 89, 90].

Normally, MAO coatings are manufactured in the oxidation reaction in electrolytes formed by NA_2_SiO_3_·9H_2_O, KOH, and KF·2H_2_O, with magnesium as the anode. The main component of the coating is magnesium oxides. The quality and chemical characteristics of MAO coatings are often influenced by the properties of the electrolyte and alloy, as well as the manufacturing process.

Figure 6 shows that in in vivo studies, compared with BM, MAO-coated Mg performs excellently in bone repair, including promoting new bone formation, increasing BV/TV, and enlarging the contact area between the bone and graft. This may result from the bubbles produced during Mg corrosion, which may inhibit physiological bone response and healing tissue formation [55]. MAO coating can also promote the material properties, which include enhancement of corrosion resistance, reduction of gas formation, and maintenance of mechanical properties during the implantation period. This degradation directly influences the mechanical strength. On the one hand, a graft with a lower degradation rate has sufficient residual volume to guarantee the mechanical strength of implants [70], whereas metal particles are produced with implant corrosion, which leads to adverse reactions, such as allergic reactions, triggering inflammation that loosens the implants [65, 71]. In addition, compared with BM, many studies have revealed that MAO-coated Mg has a better effect on the ion concentration in serum, anti-inflammation, and antiinfection, and there is no negative effect on the major organs, which indicates the great biocompatibility of MAO coatings.

However, some studies indicate that MAO coating cannot protect the alloy substrate [68, 69], which may result from the influence of surface morphology on the degradation rate. The feature morphology of the MAO coatings is a porous structure. A porous structure has both advantages and disadvantages. This structure provides a path for the electrical reaction between corrosive ions and substrates. The characteristics of pores and cracks can be seen as weak points of corrosion attack, resulting in the dense coating being corroded, which in turn exposes the Mg alloy substrate and forms a pit [68]. After the formation of pits, there is an electron effect between the coating and alloy substrate, which leads to accelerated local degradation. Therefore, the coatings were separated from the substrates when they were not completely corroded, while the BM group was protected for the accumulation of corrosion, which slows down the degradation speed [69]. Therefore, MAO coating can only protect the substrate during the limited conversion phase in the initial stage. In later immersion, degradation is accelerated owing to pores and microcracks. The pressure produced by the hydrogen and corrosion products may lead to layering. In addition, the metal compounds in the alloy may have a negative impact on the MAO coating properties, such as Mg_2_Ca in Mg-Li-Ca [21].

In addition, in contrast to most research results, the MAO-coated Mg performed poorly when guiding bone restoration in the same periods, with less new bone formed [63, 64]. The protection from MAO coating led to less Mg^2+^ release and lower concentrations. As one of the bone components, Mg^2+^ can promote the deposition of calcium salts, accelerate the formation of bone, and boost fracture healing.

### 4.2. Orthopedic Application of Fluoride-Treated Mg Alloy

Fluorine is an indispensable trace element in the human body that promotes bone growth [93]. The FLU coating is nontoxic and chemically inert [94]. The coating process is simple and involves low cost. At room temperature (37°C), the Mg alloy samples were immersed in a hydrofluoric acid solution at a specific concentration. The thickness of the coating was related to the treatment time.

Figure 6 shows that in in vivo studies, compared with BM, FLU-coated Mg performed better in bone defect repair, including promoting new bone formation and increasing bone implant contact, which may result from fluoride stimulation in osteoblastic proliferation and promotion of mineral deposition in the cancellous bone [95]. The FLU coating can also significantly improve the material properties of Mg alloys, including enhanced corrosion resistance and reduced hydrogen generation. In terms of biocompatibility, FLU coating did not affect the serum ion concentration and major organs, and no inflammatory reaction occurred.

However, FLU coating may also have shortcomings. HF is extremely toxic; it is necessary to do a good job for safety protection [95]. H_2_ is produced during HF treatment; therefore, coating is usually porous. In addition, the FLU coating thickness is insufficient to influence the desired effect [96]. These problems may be the cause of the lower performance of FLU coating compared to the other coatings.

### 4.3. Orthopedic Application of Ca-P-Coated Mg Alloys

Ca-P coatings have been widely used in bone implant surgery because of their superior biocompatibility, bioactivity, osteoinductivity, nontoxicity, and thermodynamic stability [47, 48]. HA(Ca_10_(PO_4_)_6_(OH)_2_) is the primary inorganic component of the human skeleton, which can improve the binding of the Mg matrix to the bone. After implantation, calcium and phosphorus drift off the surface of the graft and are absorbed by the body, where new tissue grows. Therefore, Ca-P has significant potential as an absorbable bone implant material [54, 97]. The coating process is simple to operate and has a high economic benefit. Mg alloy samples were prepared by immersing them in a solution of calcium salt and phosphate at a certain pH and temperature. The thickness of the coating is influenced by the treatment period. Owing to Ca/P, Mg/P, and pH, the coatings components exhibited tremendous diversity (e.g., CaHP0_4_·2H_2_O, Ca_10_(PO_4_)_6_(OH)_2_, and Ca_3_(PO_4_)_2_).

Figure 6 shows that in in vivo studies, Ca-P-coated Mg alloys performed better in bone defect repair than BM, including promoting new bone formation and improving the BV/BT and bone implant contact rate. Ca-P coating can also significantly improve the material properties, including enhanced corrosion resistance, reduced H_2_ generation, and maintenance of mechanical properties during implantation. In terms of biocompatibility, Ca-P coating did not affect serum ion concentration and major organs, and no inflammatory reaction occurred. Some studies have suggested that the Ca-P coating can improve osteoconductivity, promote differentiation of stem cells into osteoblasts, improve corrosion resistance and gas formation, and facilitate bone integration and osteoblast adhesion [44, 47]. Ca^2+^ and Mg^2+^ can also promote the proliferation of osteoblasts, inhibit bone absorption, and prevent inflammation and infection. BM was superior to Ca-P-coated Mg in terms of new bone formation during the same period, and the screw surface would be recovered with more tissue, which indicates the promotion of bone-implant contact [45]. We believe that this is due to the continuous degradation of BM and accumulation of deposition products. Under the protection of the sediment, degradation and hemolysis rates were gradually reduced. Therefore, Ca-P-coated Mg was better at new bone formation within a short period of time, but the results over longer periods of time require further observation and analysis.

The disadvantage of Ca-P coating is regulation difficulty; the formation of a specified coating requires accurate adjustments of Ca/P, Mg/P, and pH. In addition, it is very difficult to increase the content [21].

### 4.4. Previous and International Research Progress

Biodegradable Mg alloys have great clinical prospects as an ideal biomaterial for orthopedic applications. However, relevant meta-analyses and systematic reviews were limited, and previous studies simply focused on whether the Mg alloy has the same effect as materials frequently used clinically, such as titanium alloys.

Zhang et al. conducted a systematic review and meta-analysis to investigate the bone defect repair properties of biodegradable metals (pure magnesium, magnesium alloy, pure zinc, and zinc alloy) in animal models, but the results showed that the evidence quality was low, and some other biodegradable metals did not show good bone repair ability [98]. Sukotjo et al. compared the complications after osteosurgical treatment by introducing Mg or Ti screws with metal plates, and the results showed that Mg screws have a similar effect to Ti screw [99].

Sun et al. studied the effects of Mg bone implants in fracture animal models with a systematic review to affirm the positive role of Mg in bone fracture healing [100]. The lack of a standardized animal model and high heterogeneity made it difficult to perform a meta-analysis. Feeley et al. reviewed the application of different biodegradable materials in bone fixation, such as poly L-lactic acid, polyglycolide, and Mg [101]. Most Mg implants have similar clinical effects to titanium alloys, and only one study had to be removed due to severe synovitis. The effect of Mg screws in patients with immature bone growth in orthopedic surgeries was analyzed by Baldini et al. who showed that there was no negative reaction in patients using Mg screws with significant clinical effects and radiological assessment, which indicates a stable fixation effect given by Mg screws [102]. However, there have been few systematic reviews and meta-analyses of surface coatings.

### 4.5. Animal Experiments

In vitro experiments have the advantages of a short experimental cycle, easy control of experimental conditions, and good repeatability. However, in vitro experiments cannot completely simulate a complex environment, and the standards are not uniform. For example, the quality of various experimental methods cannot be evaluated, and the selection of simulated body fluids varies. Therefore, we selected animal experiments for analysis and evaluated the bone repair ability of Mg alloy implants with different coatings in vivo.

Some of the studies in this work followed the selection criteria of animal experiments, which is conducive to reducing the influence of individual differences in animals. In the selection of animal models for bone defects, 28 studies used rabbits, 12 used rats, 3 used pigs, and 1 used goat models. Rabbits and rats have been widely used in preclinical experiments. They are cheap, fast growing, easy to feed, and easy to operate. However, the bone healing and load-bearing capacity of these animals are quite different from those of humans, which may lead to deviation from clinical practice. For example, rabbits are much smaller in weight and load capacity than humans, but their bone metabolism is three times that of humans [103]. Compared with rabbits, the skeletal structure of goats is more similar to that of the human body, which is more meaningful for clinical transformation [44]. Human-sized implants can be implanted in pigs, where the soft tissue properties and wound-healing properties of the face and skull are similar to those of humans [60].

The types of surgery were mostly bone defects in the included studies. There were only five fractures, three osteotomies, and two guided bone regeneration. The extent of bone defects caused by surgery is mostly suitable for the size of implants, and different animal models have their own characteristic bone defects. Most surgeries are selected in the femur for reasons, such as simple operation and strong recovery ability. Meanwhile, as an important weight-bearing bone, the postoperative healing of the femur can be used as an important indicator of the bone repair ability of Mg alloy implants, which is of great significance for clinical application.

We conducted a comprehensive statistical analysis, which has good clinical significance and a reference value for the selection of different Mg alloy coatings. However, the final analysis may be affected to some extent by different animal models and surgical types.

### 4.6. Resources of Heterogeneity

The forest plot indicates that the comparison of new bone formation between the FLU and Ca-P-coated Mg alloys showed low heterogeneity, and the other comparisons showed high heterogeneity. This may be due to the different follow-up times, as the material degrades over time and becomes less supportive of the bone. Studies have shown that a qualified plate for fracture repair may not begin absorption until at least six weeks after surgery [104]. We included different animal species, experimental sites, chemical composition, and scaffold structure, which may have resulted in different healing rates [105]. In addition, we could not unify the variables for the Mg alloy substrates. Studies revealed that different Mg alloy substrates and Mg alloys containing different metal ratios may lead to different degradation rates and new bone formation [106–108].

### 4.7. Risk of Bias and Quality of the Evidence

The SYRCLE tool was applied to perform risk of bias assessment in the included studies [25]. Meanwhile, the CERQual and GRADE tools were applied to evaluate qualitative and quantitative evidence [27–29]. Quality assessment through these methods can be used to assess the reliability of results and the probability of future clinical transformation.

The design, process, and outcome assessment can lead to biases and, therefore, the facticity of the studies. The results demonstrate that the included studies were not of high quality. None of the included studies described their grouping process in detail, so our confidence in the overall evidence was low. Most animal studies have small sample sizes, which would result in differences in the baseline. The similarity of the baseline characteristics affects the comparability of our study. Only a small number of the studies showed similar baseline characteristics, one showed dissimilarity, and majority of the studies were not mentioned in detail. The placement of animals and whether the selection of animals is random also influences the reliability of experimental results. If animals were raised in the same environment, the nonrandom environment did not affect the results of the experiment, even though the allocation was not random [25]. Selectively reporting the animal research results can cause publication bias, which may influence the reliability of the experimental results and even produce publications with conflicting conclusions [109].

In addition, we found that the clinical transformation of the included studies was affected by implantation time, coating manufacturing parameters, individual characteristics of animal models, implant size and shape, surgical sites, and number and follow-up time of the experiments. A small number of animals also reduced our confidence in the overall evidence. We were not sure whether studies in other settings or populations would show similar results, which requires further discussion and analysis [27].

### 4.8. Limitations and Innovation

In vivo experiments were selected as research objects because of their contribution to clinical diagnosis and treatment. Conversion coatings are a popular and common coating method. To study the specificity, deposition coating and other methods have been abandoned. The authors selected different experimental sites, sample characteristics, and time groups in different studies. All these factors may have affected the results of our analysis. More than 600 articles were included in the first round of selection, and only forty-three were selected for analysis and discussion. The lack of research will be improved with the passage of time and development in the research in this field. In addition, there was no closed network in the meta-analysis in the assessment of % degradation, so we could not detect its consistency.

However, we specifically considered the coating corrosion features and their effects on new bone formation in animals for network meta-analysis. This is the first study to include material responses to the host system in qualitative outcome assessment, which will be of great significance for clinical contribution.

### 4.9. Inspiration for Future Research

Surface modification is the main method used to optimize the corrosion properties of Mg alloys. We believe that future work could focus on the following aspects to investigate perfect coatings for clinical research. First, the selection of coating materials and manufacturing technologies can be applied to promote the corrosion resistance and biocompatibility of Mg alloy implants. In terms of mechanical properties, the porosity and thickness of the coating can be a new direction for investigation. Composite coatings combined with multiple technologies have good development prospects. Second, the diversity of surgical models, different operation procedures and specific parameters of coating, various measurement methods, and data collection types lead to unsatisfactory results in this analysis and data synthesis from different studies. We hope to unify research standards to improve subsequent research. Finally, the surface modification technology of magnesium alloys is developing in the direction of environmental protection, high efficiency, and customization. In future clinical applications, different coating materials and coating methods can be selected according to different clinical requirements to achieve personalized treatment.

## 5. Conclusions

The qualitative analysis proved that MAO-coated, fluoride-treated, and Ca-P-coated Mg alloys performed excellently in orthopedic applications. These three surface coatings have excellent material properties and biocompatibility. Compared with the bare Mg alloy, the coated alloy can promote bone healing. The quantitative analysis demonstrated that MAO coating can significantly increase new bone formation, and Ca-P coating can notably enhance the corrosion resistance. Even though the included studies were of relatively low quality due to selection bias, which influenced the credibility of our results to some degree, this study is still the first one that carried out quantitative analysis on surface coatings for biomedical Mg.

## Figures and Tables

**Table 1:** Inclusion and exclusion criteria.

	Inclusion criteria	Exclusion criteria
Study design	In vivo studies including anima testing	(i) In vitro studies without animal testing (ii) Including in vitro studies only (iii) Including human clinical trials

Language	English	Other languages instead of English

Intervention	(i) Containing comparison of various magnesium alloy surface coatings (ii) Containing comparison of various coated magnesium alloys with untreated magnesium alloys	(i) Containing magnesium alloy with composite coating treatment only (ii) Containing no chemically transformed coated magnesium alloys (iii) Containing only newly synthesized composites involving Mg

Type of study	Randomized controlled trials	(i) Nonrandomized controlled trials (no control group) (ii) Studies reported only in the following forms: retrospective studies, review articles, literature reviews (iii) Publications using replicated information

Outcome	(i) Containing results related to bone healing (ii) Containing a description of the qualitative results or quantitative results of the method used to measure the properties of the material (iii) Containing assessment of material safety	(i) Only bone grafts or other materials were used (ii) Including only nonbiomechanical or histomorphology results

**Table 2:** Searching strategy and result: take Web of Science as example.

Search subject	Strategy	Result
#1 intervention	((Mg) OR (Mg alloy) OR (magnesium)) AND ((Ca-P)) OR (calcium phosphate) OR (HAP) OR (HA) OR (hydroxyapatite) OR (CaHPO_4_) OR (brushite) OR (MAO) OR (microarc oxidation) OR (microarc oxidation) OR (microarc oxidized) OR (PEO) OR (MgF_2_) OR (fluoride) OR (phytic acid) OR (PA))	87,335
#2 object of study	(in vivo) OR (animal experiment)	2,183,529
#3 type of the study	(bone fracture) OR (bone screws) OR (bone plates) OR (fracture) OR (fracture fixation) OR (bone healing) OR (bone defect) OR (bone nails)	949,809
#4 combination of all search keywords	#1, #2, AND #3	558
#5 final results with the limitation in publication date	Search filter for the publication year from 2011 to 2021	425

**Table 3:** Characteristics of the included studies.

Study ID	Animal model (control group/experimental group)								Graft materials (control group/experimental group)						Alloy substrate		Ref.
	Species	Sex	Age	Weight	Surgery type	Surgical sites	Number (animal numbers/sites numbers)		Control group			Experiment group			Model number	Element components	
							Control group	Experimental group	Graft materials	Type/shape	Sample size	Graft materials	Type/shape	Sample size			
Kong et al., 2017	Goats	Male/female	Mature	—	—	Femoral condyle; defect diameter: 4.5°mm	12 (12)	12 (12)	Uncoated JDBM	Screw	Diameter: 4.5 mm, length: 45 mm	Ca-P-coated JDBM	Screw	Diameter: 4.5 mm, length: 45 mm	JDBM	Mg (balance), Nd (3.13), Zr (0.413), Zn (0.164)	[44]
Wang et al., 2011	White rabbits	—	Adult	—	Bone defect	Femur	2 (2)	2 (2)	Uncoated AZ31B	Screw	Diameter: 5 mm, length: 3 mm	20 *μ*m Ca-P-coated AZ31B	Screw	Diameter: 5 mm, length: 3 mm	AZ31B	Mg (balance), Al (3), Zn (1)	[45]
Niu et al., 2013	New Zealand white rabbits	Male/female	Adult	2.0–2.5 kg	Bone defect	Tibia	—	—	Uncoated JDBM	Screw (Ti bone plate)	—	10–30 *μ*m Ca-P-coated JDBM	Screw (and Ti bone Plate)	—	JDBM	Mg (balance), Nd (3.13), Zr (0.413), Zn (0.164)	[46]
Han et al., 2015(a)	New Zealand white rabbits	—	Mature	—	Bone defect	Tibia; length: 10 mm	3	3	Uncoated Mg-Sr as-cast	Plate	Diameter: 0.5 mm, 2 × 3 × 10 mm^3^	5 *μ*m MAO-coated Mg-Sr as-cast	Plate	Diameter: 0.5 mm, 2 × 3 × 10 mm^3^	Mg-Sr	Mg (balance), 1.5 wt.% Sr	[58]
							3		Uncoated Mg-Sr as-extruded	Plate	Diameter: 0.5 mm, 2×3×10 mm^3^				Mg-Sr	Mg (balance), 1.6 wt.% Sr	
Sun et al., 2016	Japanese white rabbits	Male	Adult	2–3 kg	Bone defect	Mandibular and femur	15 (30)	15 (30)	Uncoated AZ31B	Screw	Diameter: 2.0 mm, length: 7.0 mm	FLU-coated AZ31	Screw	Diameter: 2.0 mm, length: 7.0 mm	AZ31B	Mg (balance), 2.50–3.50 wt.% Al, 0.60–1.40 wt.% Zn, 0.20–1.00 wt.% Mn, 0.10 wt.% Si, 0.005 wt.% Fe, 0.05 wt.% Cu, 0.005 wt.% Ni	[35]
Jiang et al., 2017	Japanese white rabbits	—	Adult	2.8–3.2 kg	Bone defect	Femur; diameter: 3 mm	6 (12)	6 (12)	Uncoated Mg–Zn–Zr	Cylinder bar	Diameter: 3 mm, length: 10 mm	0.5 *μ*m FLU-coated Mg-Zn-Zr	Cylinder bar	Diameter: 3 mm, length: 10 mm	Mg–Zn–Zr	Mg (balance), 3 wt.% Zn, 0.5 wt.% Zr	[36]
Li et al., 2017	Japanese white rabbits	Male/female	Mature	3 ± 0.2 kg	Bone defect	Femur; diameter: 3.5 mm	6 (12)	6 (12)	Uncoated MZZ	Screw	Diameter: 3.5 mm, length: 15 mm	FLU-coated MZZ	Screw	Diameter: 3.5 mm, length: 15 mm	MZZ	Mg (96/96.2218), Zn (3.2/3.1288), Zr (0.8/0.6493)	[37]
Iglesias et al., 2015	Wistar rats	Female	3 months	Average: 200 g	Bone fracture	Femur	9 (9)	9 (9)	Uncoated AZ31	Pin	Diameter: 1 mm, length: 20 mm, average weight: 28.0 ± 0.3 mg	FLU-coated AZ31	Pin	Diameter: 1 mm, length: 20 mm, average weight: 28.0 ± 0.3 mg	AZ31	Mg (balance), 3.37 ± 0.09 t.% Al, 0.78 ± 0.04 wt.% Zn, 0.22 ± 0.01 wt.% Mn	[38]
Yang et al., 2011	New Zealand white rabbits	Male/female	Mature	2.5–3.0 kg	Bone defect	Femur; diameter: 2.5 mm	6 (6)	6 (6)	Uncoated AZ31	Screw	Diameter: 2.5 mm, length: 9 mm	Ca-P-coated AZ31	Screw	Diameter: 2.5 mm, length: 9 mm	AZ31	Mg (balance), Al (3), Zn (1)	[47]
Husak et al., 2018	Rats	Male	Mature	—/	Bone defect	Tibia; 4 × 1 × 1 mm³	6	6	Uncoated Mg alloy	Rod	4 × 1 × 1 mm^3^	HA-coated Mg alloy	Rod	4 × 1 × 1 mm^3^	Mg alloy	96.25 wt.% Mg, 1.85 wt.% Al, 0.65 wt.% Zr, 1.25 wt.% Nb	[48]
Kim et al., 2018	Sprague-Dawley rats	Male	Mature	270–280 g	Bone defect	Femur; diameter: 2 mm; depth: 7 mm	3 (6)	3 (6)	Bare Mg	Screw	Diameter: 2 mm, length: 10 mm	PEO-coated Mg	Screw	Diameter: 2 mm, length: 10 mm	High purity Mg	Mg (99.9%)	[59]
								3 (6)				HA-coated Mg	Screw	Diameter: 2 mm, length: 10 mm	High purity Mg	Mg (99.9%)	
Kim et al., 2013	New Zealand white rabbits	Male/female	12 weeks	Average: 3.5 kg	Bone defect	Tibia; diameter: 2 mm; depth: 7 mm	13 (13)	13 (13)	Bare Mg	Screw	Diameter: 2 mm, length: 7 mm	HA-coated Mg	Screw	Diameter: 2 mm, length: 7 mm	High purity Mg	Mg (99.99%)	[54]
Schaller et al., 2016	Minipigs	Male/female	30–36 months	53 ± 7 kg	Bone defect	Frontal bone	6 (30)	6 (30)	Uncoated WE43	Plate screw	40 × 5 × 0.9 / 60 × 6 × 1.5 mm³；diameter: 2 mm, length: 6 mm	Plasma electrolytic-coated WE43	Plate、Screw	40 × 5 × 0.9 / 60 × 6 × 1.5 mm³；diameter: 2 mm, length: 6 mm	WE43	Mg (balance), Y, Nd	[60]
Razavi et al., 2014 (a)	Rabbits	—	Adult	3 kg	Bone defect	Femur; the greater trochanter	1	1	Uncoated AZ91	Rod	Diameter: 3 mm, length: 6 mm	PEO-coated AZ91	Rod	Diameter: 3 mm, length: 6 mm	AZ91	Mg (balance), Al (8.63), Zn (0.59), Mn (0.17), Fe (＜0.05), Cu (＜0.05)	[61]
Razavi et al., 2014 (b)	Rabbits	—	Adult	3 kg	Bone defect	Femur; the greater trochanter	—	—	Uncoated AZ91	Rod	Diameter: 3 mm, length: 6 mm	MAO-coated AZ91	Rod	Diameter: 3 mm, length: 6 mm	AZ91	Mg (balance), Al (8.63), Zn (0.59), Mn (0.17), Fe (＜0.05), Cu (＜0.05)	[62]
Lim et al., 2016	New Zealand white rabbits	Male	7 weeks	2.8–3.2 kg	Bone defect	Tibia; diameter: 2.1 mm	8 (16) right tibia	8 (16) left tibia	Uncoated WE43	Screw	Diameter: 2.3 mm, length: 5.5 mm, head diameter: 3.5 mm	HA-coated WE43	Screw	Diameter: 2.3 mm, length: 5.5 mm, head diameter: 3.5 mm	WE43	Mg (balance), 3.78 wt.% Y, 2.13 wt.% Nd, 0.46 wt.% Zr	[49]
Lim et al., 2017	Sprague-Dawley rats	Male	10 weeks	0.3–0.35 kg	Bone defect	Calvaria	25 (25)	30 (30)	Bare Mg	Plate	55.26 × 6 × 2 mm^3^	HA-coated Mg	Plate	55.26 × 6 × 2 mm^3^	High purity Mg	Mg (99.99%)	[56]
Zhuang et al., 2016	New Zealand white rabbits	Male/female	5 months	2.5–3.0 kg	Bone fracture	Radius	12 (24)	24 (36)	Bare AZ31	Strip	30×3×1 mm^3^	MAO-coated AZ31	Strip	30×3×1 mm^3^ (10 *μ*m coating, 20 *μ*m coating)	AZ31	Mg (balance), Al (2.5–3), Zn (0.7–1.3), Mn (＞ 0.2)	[63]
Wu et al., 2017	New Zealand white rabbits	Male/Female	Mature	2.5–3.0 kg	Bone fracture	Radius; width: 3 mm	6 (12)	6 (12)	Uncoated AZ31	Plate	3 × 30 × 2 mm^3^	MAO-coated AZ31	Plate	3 × 30 × 2 mm^3^ (10 *μ*m coating, 20 *μ*m coating)	AZ31	Mg (balance), Al (3.0–3.2), Zn (0.8–1.2), Mn (0.2)	[64]
								6 (12)									
Wang et al., 2020	Sprague-Dawley rats	Male	—	250 g	Bone defect	Femur; diameter: 3 mm	—	10 (20)	—			FLU-coated Mg	Scaffold	3 × 5 mm^2^	High purity Mg	Mg purity ≥ 99.98 wt.%	[43]
								10 (20)				DCPD-coated Mg	Scaffold	3 × 5 mm^2^	High purity Mg	Mg purity ≥ 99.98 wt.%	
								10 (20)				FLU-coated JDBM	Scaffold	3 × 5 mm^2^	JDBM	Mg (balance), Nd, Zn, Zr	
								10 (20)				DCPD JDBM	Scaffold	3 × 5 mm^2^	JDBM	Mg (balance), Nd, Zn, Zr	
Han et al., 2015 (b)	New Zealand white rabbits	Male	4 months	—	Bone defect	Femur	—	—	Uncoated Mg-Sr	Rod	Diameter: 2 mm, length: 6 mm	Sr-Ca-P contained MAO-coated Mg-Sr	Rod	Diameter: 2 mm, Length: 6 mm	Mg–Sr	Mg (balance), 1.5 wt.% Sr	[65]
Xiao et al., 2013	New Zealand white rabbits	Male/female	Adult	2.5–3.0 kg	Bone defect	Femur; shafts lengthened 8 mm	12 (24)	12 (24)	Uncoated AZ60	Plate	Diameter: 3 mm, length: 8 mm	Ca-P-coated AZ60	Plate	Diameter: 3 mm, Length: 8 mm	AZ60	Mg (balance), Al (5.8–7.2), Zn (<1.0), Mn (0.15–0.5), Si (0.1), Cu (0.05), Ni (0.005), Fe (0.005)	[57]
Sun et al., 2013	Japanese White rabbits	Male/female	—	2.8–3.0 kg	Bone defect	Femur; shafts lengthened 3 mm	3 (6)	3 (6)	Uncoated Mg-Zn-Zr	Rod	Diameter: 3 mm, length: 10 mm	Ca-P-coated Mg-Zn-Zr	Rod	Femoral shaft: 3 mm	Mg-Zn-Zr	Mg (balance), Zn (3), Zr (0.8)	[39]
								3 (6)				FLU-coated Mg-Zn-Zr	Rod	Femoral shaft: 4 mm			
Wu et al., 2019	New Zealand white rabbits	Male/female	Mature	2.5–3.0 kg	Bone defect	Ulna; width: 15 mm	12 (24)	12 (24)	Uncoated Mg alloy	Scaffold	Inside diameter: 3 mm, outside diameter: 5 mm, length: 15 mm	MAO-coated Mg	Scaffold	Inside diameter: 3 mm, outside diameter: 5 mm, length: 15 mm (10 *μ*m coating, 20 *μ*m coating)	Mg alloy	Mg (balance), Zn (2.6), Ca (1.5), Al (0.2), Ce (0.1), P (0.6), Gd (0.2)	[66]
								12 (24)									
Xu et al., 2018	Sprague-Dawley rats	Male	—	150–170 g	Bone defect	Femur; distal femur condyles; diameter: 2.5 mm	6	6	Bare Mg	Rod	—	SO-coated Mg	Rod	—	High purity Mg	Mg (99.9%)	[67]
								6				MAO-coated Mg	Rod				
Fischerauer et al., 2012	Sprague-Dawley rats	Male	25 weeks	140–160 g	Bone defect	Femur	10 (20)	10 (20)	Uncoated ZX50	Pin	—	MAO-coated ZX50	Pin	—	ZX50	Mg (balance), 5 wt.% Zn, 0.25 wt.% Ca, 0.15 wt.% Mn	[68]
Lin et al., 2013	White rabbits	—	5 weeks	1.5–2 kg	Bone defect	Femur; mid-diaphyseal region; diameter: 2 mm	9 (18)	9 (18)	Uncoated ZK60	Rod	Diameter: 2 mm, length: 6 mm	MAO-coated ZK60	Rod	Diameter: 2 mm, length: 6 mm (10 *μ*m coating)	ZK60	Mg (balance), 5.5 wt.% Zn, 0.4 wt.% Zr	[69]
Wang et al., 2020	Sprague-Dawley rats	Male	Adult	—	Bone defect	Femur; shafts lengthened 10 mm；diameter：2 mm	4 (4)	4 (4)	Bare Mg	Cylindrical implant	Diameter: 2 mm, length: 10 mm	PEO-coated Mg	Cylindrical implant	Diameter: 2 mm, length: 10 mm (5.12 ± 0.37 *μ*m coating)	High purity Mg	Mg purity > 99.95 wt.%	[70]
Song et al., 2019	New Zealand white rabbits	—	6 weeks	800–1200 g	Bone defect	Femur; diameter: 1.3 mm	18 (18)	18 (18)	Uncoated Mg-Ca-Zn	Pin	Diameter: 1.3 mm, length: 35 mm	PEO-coated Mg-Ca-Zn	Pin	Diameter: 1.3 mm, length: 35 mm	Mg-Ca-Zn	94 wt.% Mg, 5 wt.% Ca, 1 wt.% Zn	[11]
Barbeck et al., 2020	New Zealand white rabbits	Female	12 weeks	2.2–2.9 kg	Bone defect	Skull; diameter: 8 mm	18 (36)	18 (36)	Uncoated AZ31	Mesh	30 × 40 mm	HF-treated AZ31	Mesh	30 × 40 mm	AZ31	—	[40]
Chai et al., 2011	Wistar rats	—	—	—/	Bone defect	Femur; diameter: 1 mm	3 (3）	3 (3)	Uncoated AZ31	Rod	Diameter: 1 mm, length: 5 mm	85 *μ*m *β*-TCP-coated AZ31	Rod	Diameter: 1 mm, length: 5 mm	AZ31	Ca, P, Zn, Mg	[50]
Razavi et al., 2014 (c)	Rabbits	—	Adult	—	Bone defect	Femur; the greater trochanter；diameter：3 mm	—	—	Uncoated AZ91	Rod	Diameter: 3 mm, length: 6 mm	MAO-coated Mg	Rod	Diameter: 3 mm, length: 6 mm (100 *μ*m coating)	AZ91	—	[71]
Bai et al., 2017	New Zealand white rabbits	Male	3 months	2.2-2.3 kg	Bone defect	Femur; the greater trochanter；diameter：3.2 mm	12 (24)	12 (24)	Uncoated	Column	Diameter: 3.2 mm, length: 12 mm	MAO-coated	Column	Diameter: 3.2 mm, length: 12 mm	None	—	[72]
Smith et al., 2011	Rabbits	—	—	—	Bone defect	Ulna; length: 15 mm	5 (5)	2 (2)	—	—	—	Ca-P-coated AZ31	Cylinder	Diameter: 3.5 mm, length: 12 mm	AZ31	—	[51]
Naujokat et al., 2019	Pigs	—	Average: 10 months	22–24 kg	Osteotomy; bone defect	Mandibular; width 0.35 mm, length 10 mm, depth 6 mm	3 (6)	3 (6)	Uncoated MgYREZr	Plate and screw	Thickness: 1 mm, length: 22 mm and diameter: 2 mm, length: 5 mm	FLU-coated MgYREZr	Plate and screw	Thickness: 1 mm, length: 22 mm and diameter: 2 mm, length: 5 mm	MgYREZr	Mg (> 90), Y, Zr	[41]
Schaller et al., 2017	Minipigs	—	30–36 months	53 ± 7 kg	Osteotomy; bone defect	Rib; the 7th–9th ribs	6 (12)	6 (12)	Uncoated WE43	Plate and screw	47 × 7 × 1.8 mm^3^ and diameter: 2 mm, length: 6 mm	PEO-coated WE43	Plate and screw	47 × 7 × 1.8 mm and diameter: 2 mm, length: 6 mm	WE43	Mg (balance), Y, Nd	[76]
Razavi et al., 2020	Rabbits	—	Adult	Average: 3 kg	Bone defect	Femur; the greater trochanter；diameter：3 mm	—	—	Uncoated AZ91	Rod	—	MAO-coated AZ91	Rod	—	AZ91	Mg (balance), Al (9), Zn (1), Mn (0.2), Fe (< 0.005)	[55]
Liu et al., 2019	Sprague-Dawley rats	Male	—	250 ± 10 g	Bone defect	Femur; diameter: 2 mm	—	—	Uncoated AZ91	Cylinder	Diameter: 2 mm, length: 5 mm	MAO-coated AZ91	Cylinder	Diameter: 2 mm, Length: 5 mm	AZ91	—	[73]
Wu et al., 2021	Sprague-Dawley rats	Male	8 weeks	250 ± 20 g	Guided bone regeneration	Calvaria; diameter: 8 mm	8 (8)	8 (8)	Bare Mg	Mesh	Diameter: 10 mm, hole diameter: 0.4 mm	Ca-P-coated Mg	Mesh	Diameter: 10 mm, Hole Diameter: 0.4 mm	High purity Mg	Mg (99.9%)	[52]
Bodelón et al., 2015	Wistar rats	Female	3 months	200 g	Bone fracture	Femur	9 (9)	9 (9)	Uncoated AZ31	Pin	Diameter: 1 mm, length: 20 mm, average weight: 28.0 ± 0.3 mg	FLU-coated AZ31	Pin	Diameter: 1 mm, length: 20 mm, average weight: 28.0 ± 0.3 mg	AZ31	Mg (balance), 3.37 ± 0.09 wt.% Al, 0.78 ± 0.04 wt.% Zn, 0.22 ± 0.01 wt.% Mn	[42]
Razavi et al., 2015	Rabbits	—	Adult	3 kg	Bone defect	Femur; the greater trochanter; diameter: 3 mm	1 (1)	1 (1)	Uncoated AZ91	Rod	Diameter: 3 mm, length: 6 mm	PEO-coated AZ91	Rod	Diameter: 3 mm, length: 6 mm	AZ91	Mg (balance), 8.63 wt.% Al, 0.59 wt.% Zn, 0.17 wt.% Mn, 0.05 wt.% Fe, 0.05 wt.% Cu	[74]
Zhang et al., 2018	New Zealand white rabbits	Male/female	6.23 ± 0.37 months	2.56 ± 0.25 kg	Osteotomy; bone defect	Ulna; 15 mm apart	18 (36)	18 (36)	Uncoated Mg-Zn-Ca	Scaffold	Inside diameter: 3 mm, outside diameter: 5 mm, hole diameter: 1 mm, length: 15 mm	MAO-coated Mg-Zn-Ca	Scaffold	Inside diameter: 3 mm, outside diameter: 5 mm, hole diameter: 1 mm, length: 15 mm (10 *μ*m coating)	Mg-Zn-Ca	Mg (0.5), Zn (2.5–3.0), Ca (0.5–1.5)	[75]
Peng et al., 2019	New Zealand white rabbits	Male	6 months	2.5–3.0 kg	Guided bone regeneration	Calvaria; diameter: 6 mm; depth: 1.3 mm	9 (9)	9 (9)	Bare Mg	Square membrane	Thickness: 50 *μ*m, width: 8 mm	Ca-P-coated Mg	Square membrane	Thickness: 50 *μ*m, width: 8 mm	High purity Mg	Mg purity >99.95%	[53]

**Table 4:** Processing parameters of MAO coatings.

Study ID	Ref.	Coating components	Solution	Specific parameter					
				Temperature (°C)	Time (min)	Work voltage (V)	Work frequency (Hz)	Work duty cycle (%)	Current
Han et al., 2015 (a)	[58]	8 g/L KF·2H_2_O, 4 g/L (NaPO_3_)_6_, 0.8 g/L Ca(OH)_2_, 0.8 g/L Sr(OH)_2_	**—**	20–25	5	360	1000	40	**—**
Kim et al., 2018	[59]	1.0 M NaOH, 0.1 M Na_3_PO_4_, 0.1 M glycerol		**—**	3	Star voltage of the pulse: 10	**—**	50	Unipolar pulse current: 300 mA/cm^2^
Schaller et al., 2016	[60]	Magoxid electrolyte			**—**	400		**—**	current: 1.4 A/dm^2^
Razavi et al., 2014 (a)	[61]	200 g/L Na_2_SiO_3_, 200 g/L NaOH			30	60			**—**
Razavi et al., 2014 (b)	[62]	200 g/L Na_2_SiO_3_, 200 g/L NaOH			30	60			
Zhuang et al., 2016	[63]	**—**			**—**	**—**			
Wu et al., 2017	[64]	MgO, Mg_2_SiO_4_, CaSiO_3_, Mg_3_(PO_4_)_2_	10 g/L Ca(H_2_PO_4_)_2_, 15 g/L Na_2_SiO_3_, 10 g/L NaOH	<40	5	400	600	8	
					10				
Han et al., 2015 (b)	[65]	MgO, MgF_2_	8 g/L KF·2H_2_O, 4 g/L (NaPO_3_)_6_, 0.8 g/L Ca(OH)_2_, 0.8 g/L Sr(OH)_2_	20−25	5	360	1000	40	
Wu et al., 2019	[66]	MgO, Mg_2_SiO_4_, CaSiO_3_, Mg_3_(PO_4_)_2_	10 g/L Ca(H_2_PO_4_)_2_, 15 g/L Na_2_SiO_3_, 10 g/L NaOH	<50	5	500	600	8	
					10				
Xu et al., 2018	[67]	MgO	12 g/L Na_2_SiO_3_·9H_2_O, 2 g/L (NaPO_3_)_6_ **—**	**—**	2	300	**—**	**—**	
Fischerauer et al., 2012	[68]	**—**	Saline		**—**				Current density: 14 mA/cm^2^
						**—**			
Lin et al., 2013	[69]	MgO, Mg_2_SiO_4_	10 g/L Na_2_SiO_3_·9H_2_O, 1 g/L KOH, 8 g/L KF·2H_2_O			300	1000	40	**—**
Wang et al., 2020	[70]	MgO, Mg_2_SiO_4_	0.04 M Na_2_SiO_3_·9H_2_O, 0.1 M KOH, 0.2 M·KF·2H_2_O			360	800	10	Constant current: 0.8 A
Song et al., 2019	[11]	**—**	**—**			**—**	**—**	**—**	**—**
Razavi et al., 2014 (c)	[71]	Mg, MgO, Mg_2_SiO_4_	200 g/L Na_2_SiO_3_, 200 g/L NaOH		30	60			
Bai et al., 2017	[72]	MgO	**—**		10	450	100	26	Current: 400 A
Schaller et al., 2017	[76]	Mg_3_(PO_4_)_2_, some traces of yttrium and neodymium	Magoxid electrolyte		**—**	400	**—**	**—**	Current: 1.4 A/dm^2^
Razavi et al., 2020	[55]	MgO, Mg_2_SiO_4_	200 g/L NaOH, 200 g/L Na_2_SiO_3_		30	60			**—**
Liu et al., 2019	[73]	**—**	0.04 M/L NaH_2_PO_4_, 0.1 M/L Ca(CH_3_COO)_2_		3	450			
Razavi et al., 2015	[74]		200 g/L Na_2_SiO_3_, 200 g/L NaOH		30	60			
Zhang et al., 2018	[75]	MgO, Mg_2_SiO_4_, Mg(OH)_2_, CaSiO_3_	10 g/L Ca(H_2_PO_4_)_2_, 15 g/L Na_2_SiO_3_, 10 g/L NaOH	30	**—**	450	600	8	

**Table 5:** Ca-P and FLU treatment manufacturing parameters.

Study ID	Ref.	Type	Coating components	Specific parameter			
				Solution	pH value	Temperature (°C)	Time
Niu et al., 2013	[46]	Ca-P coating	CaHPO_4_·2H_2_O	0.1 M KF	—	—	24 h
				NaNO_3_, Ca(H_2_PO_4_)_2_·H_2_O, H_2_O_2_	—	20	24 h
Yang et al., 2011	[45]		Mg(H_2_PO_4_)_2_, Ca(H_2_PO_4_)_2_, Ca_3_(PO_4_)_2_, Mg_3_(PO_4_)_2_	Ca(NO_3_)_2_, NH_4_H_2_PO_4_	4	60	24 h
Niu et al., 2013	[46]		CaHPO_4_·2H_2_O	0.1 M KF	—	—	24 h
				NaNO_3_, Ca(H_2_PO_4_)_2_·H_2_O, H_2_O_2_		20	24 h
Yang et al., 2011	[47]		Ca_10_(PO_4_)_6_(OH)_2_	Na_2_HPO_4_·12H_2_O, NaHCO_3_, Ca(NO_3_)_2_·4H_2_O		37	24 h
Husak et al., 2018	[48]		Ca_10_(PO_4_)_6_(OH)_2_	0.05 M CaCl_2_, 0.03 M Na_2_HPO_4_		30	1.5 h
Kim et al., 2013	[54]		Ca_10_(PO_4_)_6_(OH)_2_	0.05 M C_10_H_12_CaN_2_Na_2_O_8_, 0.05 M KH_2_PO_4_	8.9	90	2 h
Lim et al., 2016	[49]		Ca_10_(PO_4_)_6_(OH)_2_	0.05 M C_10_H_12_CaN_2_Na_2_O_8_, 0.05 M KH_2_PO_4_	8.9	90	2 h
Lim et al., 2017	[56]		Ca_10_(PO_4_)_6_(OH)_2_	0.05 M C_10_H_12_CaN_2_Na_2_O_8_, 0.05 M KH_2_PO_4_	8.9	90	2 h
Wang et al., 2020	[43]		CaH_2_PO_4_·2H_2_O	60 g/L NaNO_3_, 15 g/L Ca(H_2_OP_4_)_2_·H_2_O, 20 mL/L 30 wt.% H_2_O_2_	—	—	—
Xiao et al., 2013	[57]		CaHPO_4_·2H_2_O, Mg_3_(PO_4_)_2_	—	2.6–2.8	37 ± 2	30 min
Sun et al., 2013	[39]		—	0.15 M KH PO_4_, 0.15 M CaCl_2_	—	Room temperature	4 d
Chai et al., 2011	[50]		*β*-Ca_3_(PO_4_)_2_	Supersaturated Na_2_HPO_4_		Room temperature	3 h
				23.75 g/L Na_2_HPO_4_·12H_2_O, 18.2 g/L Ca(NO_3_)_2_		70	48 h
Smith et al., 2011	[51]		Ca_3_(PO_4_)_2_, Mg	5 mM NaOH		—	24 h
				1.5 mM MgCl_2_, 1.5 mM CaCl_2_, 1.8 mM Na_2_HPO_4_		—	12 d
Wu et al., 2021	[52]		—	0.25 M C_10_H_12_CaN_2_Na_2_O_8_, 0.25 M KH_2_PO_4_	8.9	90	2 h
Peng et al., 2019	[53]		Ca_3_(PO_4_)_2_	0.1 M KF	—	20	24 h
				NaNO_3_, Ca(H_2_PO_4_)_2_·H_2_O, H_2_O_2_			24 h
Sun et al., 2016	[35]	FLU coating	MgO, MgF_2_	50 wt.% HF		30	72 h
Jiang et al., 2017	[36]		MgF_2_	20% HF		37	6 h
Li et al., 2017	[37]		MgF_2_, Mg	20% HF		37	12 h
Iglesias et al., 2015	[38]		MgF_2_	48 wt.% HF		Room temperature	24 h
Wang et al., 2020	[43]		MgF_2_	40 wt.% HF		Room temperature	24 h
Sun et al., 2013	[39]		MgF_2_	20% HF		37	6 h
Barbeck et al., 2020	[40]		MgF_2_	—		—	—
Naujokat et al., 2019	[41]		MgF_2_	F_2_ activation with NaOH		—	—
Bodelón et al., 2015	[42]		MgF_2_	48 wt.% HF		Room temperature	24 h

**Table 6:** Evidence of the qualitative analysis using CERQual analysis.

Outcome		Number of included studies	Methodological limitations	Correlation	Coherence	Adequacy	Quality of the evidence (CERQual)
Bone repair	New bone formation	38	Selection bias; performance bias; attrition bias; other biases	The included studies were relevant to the review in terms of background, aim, interventions, and intervention subjects. However, the clinical transformation was affected by various factors, including follow-up time, coating processing technology, implant design, animal model, in vivo environment differences, object characteristics, sample size, defect size, and positions.	Out of the included 38 research studies, 23 studies revealed that in terms of new bone formation, the intervention groups (coated group) were better than the control group. However, four studies reported the opposite result. Meanwhile, five studies showed that there is no significant difference between two groups. There was no comparison between two groups in eight studies.	New bone formation was assessed using both qualitative and quantitative analyses.	⊖⊖⨁⨁low
	BV/TV	6	Selection bias; performance bias; other biases	The included studies were relevant to the review in terms of background, aim, interventions, and intervention subjects. However, the clinical transformation was affected by various factors, including follow-up time, coating processing technology, implant design, animal model, in vivo environment differences, object characteristics, sample size, defect size, and positions.	Out of the six studies included, four studies revealed that in terms of the value of BV/TV, the intervention groups (coated group) were higher than the control group. However, the reverse result was reported in another study. Meanwhile, there was no comparison between two groups in another study.	BV/TV was assessed quantitatively.	⊖⊖⨁⊖very low
	Bone-implant contact	8	Selection bias; performance bias; other biases	The included studies were relevant to the review in terms of background, aim, interventions, and intervention subjects. However, the clinical transformation was affected by various factors, including follow-up time, coating processing technology, implant design, animal model, in vivo environment differences, object characteristics, sample size, defect size, and positions.	Out of the eight studies included, three studies revealed that in terms of bone-implant contact, the intervention groups (coated group) had closer contact than the control group. Meanwhile, only one study showed that there is no significant difference between two groups. There was no comparison between two groups in three studies.	Bone-implant contact was assessed using both qualitative and quantitative analysis.	⊖⊖⨁⊖very low
Material properties	Degradation	39	Selection bias; performance bias; attrition bias; other biases	The included studies were relevant to the review in terms of background, aim, interventions, and intervention subjects. However, the clinical transformation was affected by various factors, including follow-up time, coating processing technology, implant design, animal model, in vivo environment differences, object characteristics, sample size, defect size, and positions.	Out of the included 39 researches, 34 studies revealed that in terms of degradability, the intervention groups (coated group) corroded slower than control groups and have better complete shape. However, three studies reported the opposite result. Meanwhile, three studies showed that there is no significant difference between two groups. There was no comparison between two groups in another study.	Degradability was assessed using both qualitative and quantitative analyses.	⊖⊖⨁⨁low
	Gas formation	27	Selection bias; performance bias; other biases	The included studies were relevant to the review in terms of background, aim, interventions, and intervention subjects. However, the clinical transformation was affected by various factors, including follow-up time, coating processing technology, implant design, animal model, in vivo environment differences, object characteristics, sample size, defect size, and positions.	Out of the included 27 research studies, 15 studies revealed that in terms of gas formation, the intervention groups (coated group) had less hydrogen generated than the control group. However, seven studies reported the opposite result. Meanwhile, another study showed that there is no significant difference between two groups. There was no comparison between two groups in six studies.	Gas formation was assessed using both qualitative and quantitative analyses.	⊖⊖⨁⨁low
	Mechanical properties	4	Selection bias; performance bias; other biases	The included studies were relevant to the review in terms of background, aim, interventions, and intervention subjects. However, the clinical transformation was affected by various factors, including follow-up time, coating processing technology, implant design, animal model, in vivo environment differences, object characteristics, sample size, defect size, and positions.	Out of the four studies included, three studies showed that the mechanical properties were better in the intervention groups (coated groups). There was no comparison between two groups in other studies.	Mechanical properties were assessed quantitatively.	⊖⊖⨁⊖very low
Systemic host response	Influence in the major organs	8	Selection bias	The included studies were relevant to the review in terms of background, aim, interventions, and intervention subjects. However, the clinical transformation was affected by various factors, including follow-up time, coating processing technology, implant design, animal model, in vivo environment differences, object characteristics, sample size, defect size, and positions.	Out of the eight studies included, one study showed that intervention groups (coated groups) had less influence on the major organs. Seven studies demonstrated that there was no significant difference between the two groups. There was no comparison between the two groups in two other studies.	The influence on major organs was assessed using both qualitative and quantitative analyses.	⊖⊖⨁⊖very low
	Ions concentration in serum	13	Selection bias; performance bias; attrition bias; other biases	The included studies were relevant to the review in terms of background, aim, interventions, and intervention subjects. However, the clinical transformation was affected by various factors, including follow-up time, coating processing technology, implant design, animal model, in vivo environment differences, object characteristics, sample size, defect size, and positions.	Out of the 13 studies included, nine studies found that the intervention groups have less influence on the concentration of ions in serum than the control group. Meanwhile, five studies demonstrated that there is no significant difference between two groups and there is no comparison between the two groups in other studies.	The concentration of ions in serum before and after implantation was assessed quantitatively.	⊖⊖⨁⨁low
	Clinical findings and infectious	24	Selection bias; performance bias; attrition bias; other biases	The included studies were relevant to the review in terms of background, aim, interventions, and intervention subjects. However, the clinical transformation was affected by various factors, including follow-up time, coating processing technology, implant design, animal model, in vivo environment differences, object characteristics, sample size, defect size, and positions.	Out of the 24 studies included, nine studies demonstrated that the intervention groups have better antiinfectious ability. Four studies revealed that there was no significant difference between the intervention groups and control groups, and there were no comparisons between the two groups in another 13 studies.	Clinical infections were assessed qualitatively.	⊖⊖⨁⨁low

**Table 7:** Evidence of quantitative results using GRADE analysis.

Outcome measurement	Number of included studies	Study limitations in risk of bias	Indirectness	Inconsistency	Imprecision	Publication bias (h)	Quality of the evidence (GRADE)
% degradation	16	−1^a^	−1^c, d^	−1^e^	−1^g^	−1	⊕⊝⊝⊝ very low
New bone formation	8	−1^a, b^	−1^c, d^	−1^e, f^	−1^g^	−1	⊕⊝⊝⊝ very low

^a^There was no randomly generated and fully concealed allocation sequence; participants were not blinded; there were other biases. ^b^Baseline characteristics were not similar and the result data were incomplete. ^c^There are differences between experimental designs. (including the number and type of animals and surgical scheme, types of coatings and magnesium alloys, implant design, and implantation time). ^d^There is indirectness between the research object (animal) and the clinical transformation object (human). ^e^The heterogeneity test value (*I*^2^) and funnel plot indicate that there is high heterogeneity and the point estimates vary greatly. ^f^The consistency check result (chi^2^ (1) = 0.63, *P* = 0.428) shows that there is no inconsistency. ^g^The confidence interval contains invalid values, and the ranking of interventions varies greatly and is not easy to change. ^h^The funnel diagram shows a high degree of asymmetry.

## Data Availability

The data used to support the findings of this research are included within the article and are labeled with references.

## Supplementary Materials

Supplementary Materials Figure S1. Contribution graph of % degradation. Figure S2. Contribution graph of new bone formation. Table S1. Searching strategy and result on the PubMed. Table S2. Searching strategy and result on Science Direct. Table S3. Searching strategy and result on Web of Science. Table S4. Included and excluded studies after first screening.
