# Supplementary material for: A Systematic Review and Network Meta-Analysis of Biomedical Mg Alloy and Surface Coatings in Orthopedic Application
Source: Bioinorg Chem Appl. 2022 Mar 31;2022:4529520. doi: 10.1155/2022/4529520 (PMC8991394; doi:10.1155/2022/4529520)
Supplement: Supplementary Materials — Figure S1. Contribution graph of % degradation. Figure S2. Contribution graph of new bone formation. Table S1. Searching strategy and result on the PubMed. Table S2. Searching strategy and result on Science Direct. Table S3. Searching strategy and result on Web of Science. Table S4. Included and excluded studies after first screening. [file 4529520.f1.zip › 4529520.f1/(Supplementary materials) Figure S1-S2.pptx]

## Slide 1
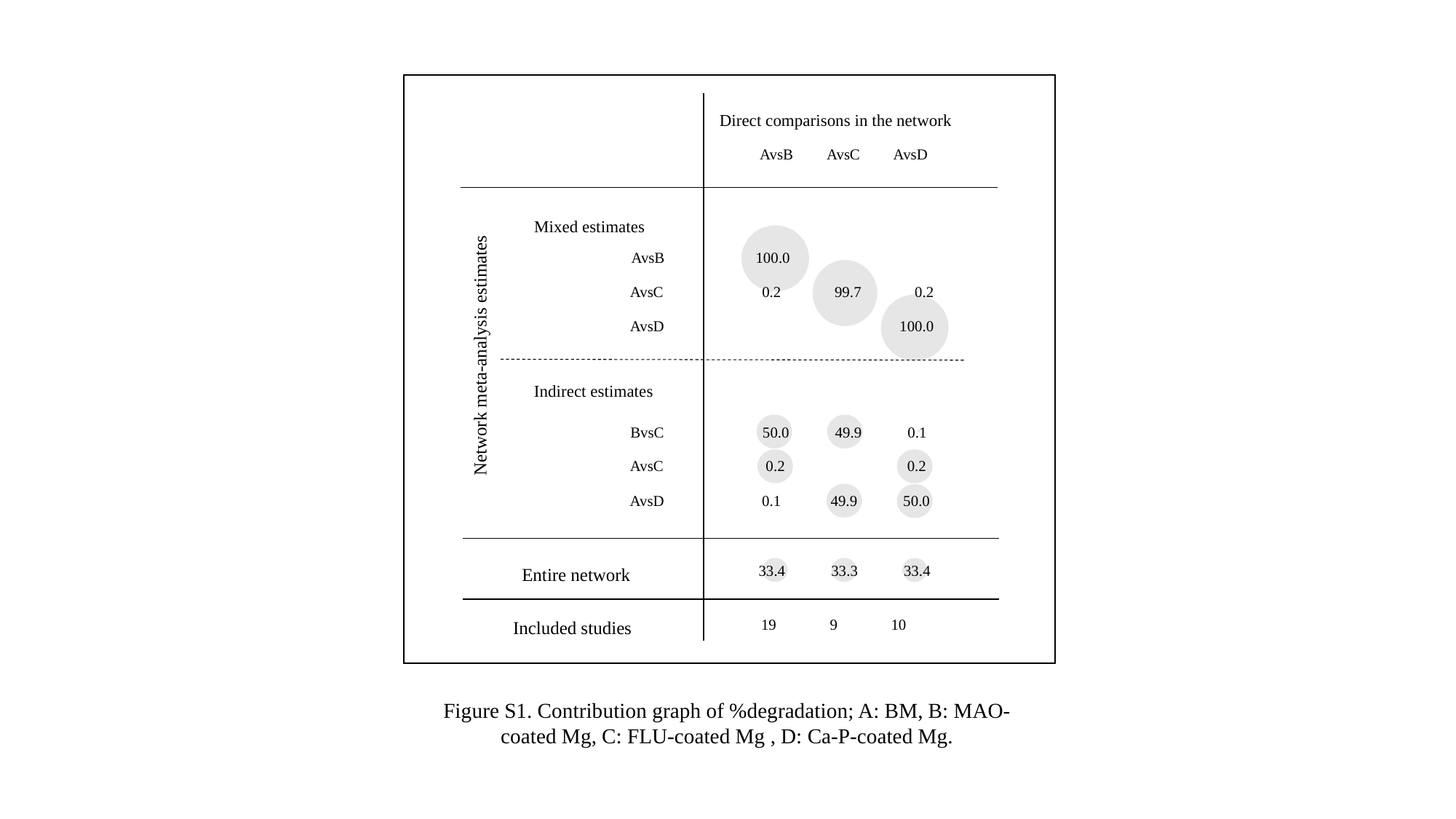

Direct comparisons in the network
AvsB AvsC AvsD
Mixed estimates
·
·
 33.4 33.3 33.4
AvsB 	 100.0
·
·
AvsC	 0.2 99.7 0.2
AvsD	 100.0
Network meta-analysis estimates
Indirect estimates
BvsC	 50.0 49.9 0.1
AvsC	 0.2 0.2
AvsD	 0.1 49.9 50.0
Entire network
Included studies
 19 9 10
Figure S1. Contribution graph of %degradation; A: BM, B: MAO-coated Mg, C: FLU-coated Mg , D: Ca-P-coated Mg.

## Slide 2
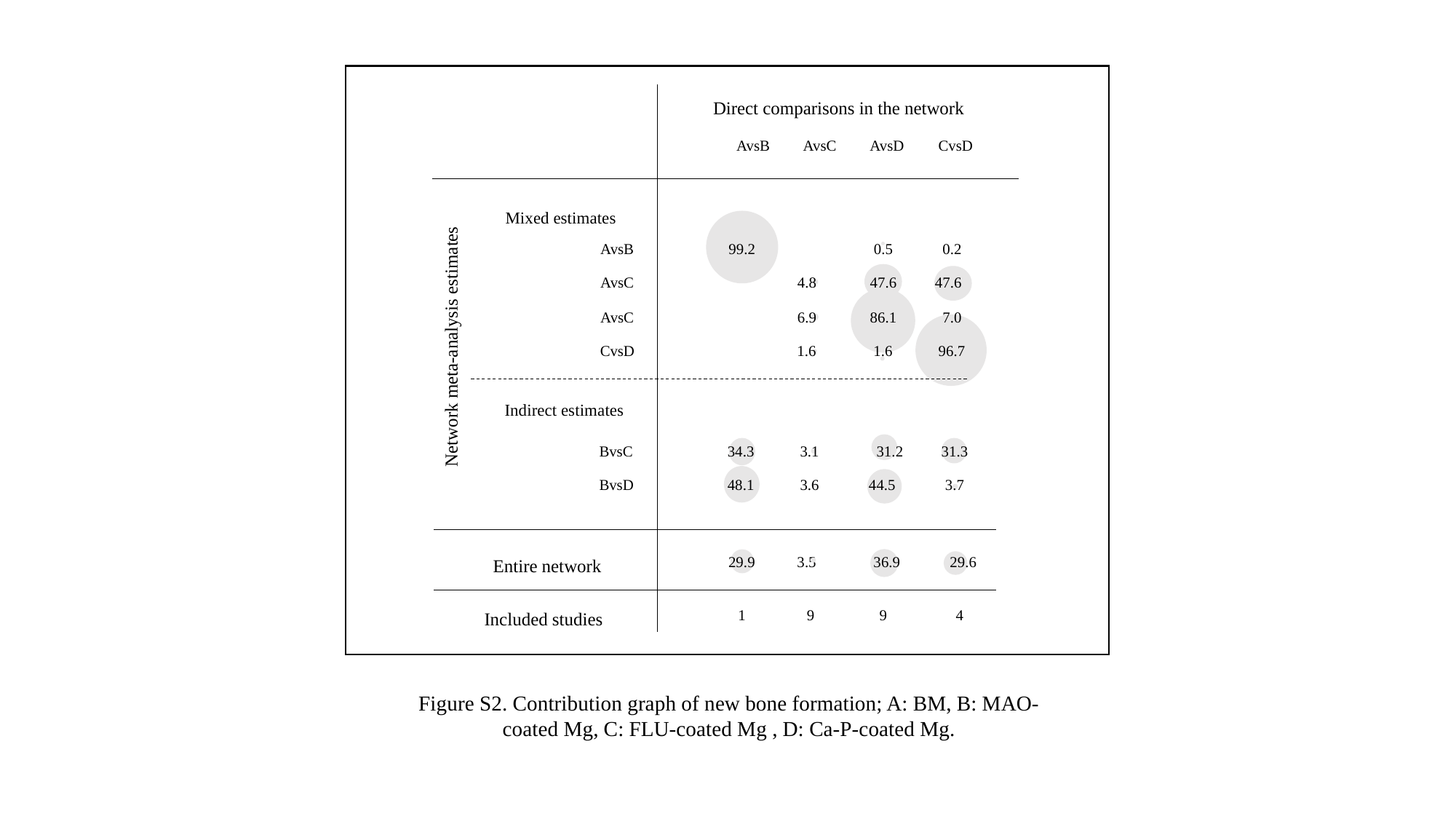

Direct comparisons in the network
AvsB AvsC AvsD CvsD
Mixed estimates
AvsB 	 99.2 0.5 0.2
AvsC 	 4.8 47.6 47.6
AvsC 	 6.9 86.1 7.0
Network meta-analysis estimates
CvsD 	 1.6 1.6 96.7
Indirect estimates
BvsC 	 34.3 3.1 31.2 31.3
BvsD 	 48.1 3.6 44.5 3.7
Entire network
 29.9 3.5 36.9 29.6
Included studies
 1 9 9 4
Figure S2. Contribution graph of new bone formation; A: BM, B: MAO-coated Mg, C: FLU-coated Mg , D: Ca-P-coated Mg.
