# Supplementary material for: A Systematic Review and Network Meta-Analysis of Biomedical Mg Alloy and Surface Coatings in Orthopedic Application
Source: Bioinorg Chem Appl. 2022 Mar 31;2022:4529520. doi: 10.1155/2022/4529520 (PMC8991394; doi:10.1155/2022/4529520)
Supplement: Supplementary Materials — Figure S1. Contribution graph of % degradation. Figure S2. Contribution graph of new bone formation. Table S1. Searching strategy and result on the PubMed. Table S2. Searching strategy and result on Science Direct. Table S3. Searching strategy and result on Web of Science. Table S4. Included and excluded studies after first screening. [file 4529520.f1.zip › 4529520.f1/(Supplementary materials) Tables S4.docx]

**Table S4. Included and Excluded studies after first screening**

| **Nr** | **Paper** | | | **Status** |
| --- | --- | --- | --- | --- |
|  | **Authors** | **Article Title** | **Publication Year** |  |
| 1 | Guoyuan Li, Lei Zhang, Lei Wang, Guangyin Yuan, Kerong Dai, Jia Pei, Yongqiang Hao | Dual modulation of bone formation and resorption with zoledronic acid-loaded biodegradable magnesium alloy implants improves osteoporotic fracture healing: an in vitro and in vivo study | 2017 | Excluded; graft not applied in orthopedic |
| 2 | M. Bornapour, H. Mahjoubi, H. Vali, D. Shum-Tim, M. Cerruti, M. Pekguleryuz | Surface characterization, in vitro and in vivo biocompatibility of Mg-0.3Sr-0.3Ca for temporary cardiovascular implant | 2016 | Excluded; graft not applied in orthopedic |
| 3 | Shaylin Shadanbaz, Jemimah Walker, Tim B. F. Woodfield, Mark P. Staiger, George J. Dias | Monetite and brushite coated magnesium: in vivo and in vitro models for degradation analysis | 2013 | Excluded; graft not applied in orthopedic |
| 4 | Yongseok Jang, Zongqing Tan, Chris Jurey, Boyce Collins, Aditya Badve, Zhongyun Dong, Chanhee Park, Cheol Sang Kim, Jagannathan Sankar, Yeoheung Yun | Systematic understanding of corrosion behavior of plasma electrolytic oxidation treated AZ31 magnesium alloy using a mouse model of subcutaneous implant | 2014 | Excluded; graft not applied in orthopedic |
| 5 | Benoît Schaller, John Patrik Matthias Burkhard, Madeleine Chagnon, Stefan Beck, Thomas Imwinkelried, Michel Assad | Fracture healing and bone remodeling with human standard-sized magnesium versus polylactide-co-glycolide (PLGA) plate and screw systems using a mini-swine craniomaxillofacial osteotomy fixation model | 2018 | Excluded; irrelevant comparators |
| 6 | Jialin Niua, Meiping Xiong, Xingmin Guana, Jian Zhanga, Hua Huanga, Jia Pei, Guangyin Yuan | The in vivo degradation and bone-implant interface of Mg-Nd-Zn-Zralloy screws: 18 months post-operation results | 2016 | Excluded; irrelevant comparators |
| 7 | Jian Tang, Jiali Wang, Xinhui Xie, Peng Zhang, Yuxiao Lai, Yangde Li, Ling Qin | Surface coating reduces degradation rate of magnesium alloy developed for orthopaedic applications | 2013 | Excluded; irrelevant comparators |
| 8 | Jiawen Si, Hongzhou Shen, Hongwei Miao, Yuan Tian, Hua Huang, Jun Shi, Guangyin Yuan, Guofang Shen | In vitro and in vivo evaluations of mg-Zn-Gd alloy membrane on guided bone regeneration for rabbit calvarial defect | 2020 | Excluded; irrelevant comparators |
| 9 | Min-Ho Kang, Hyun Lee, Tae-Sik Jang, Yun-Jeong Seong, Hyoun-Ee Kim, Young-Hag Koh, Juha Song, Hyun-Do Jung | Biomimetic porous Mg with tunable mechanical properties and biodegradation rates for bone regeneration | 2018 | Excluded; irrelevant comparators |
| 10 | N Kleer-Reiter, S Julmi, F Feichtner, A-C Waselau, C Klose, P Wriggers, H J Maier, A Meyer-Lindenberg | Biocompatibility and degradation of the open-pored magnesium scaffolds LAE442 and La2 | 2021 | Excluded; irrelevant comparators |
| 11 | N. Kleer, S. Julmi, A.-K. Gartzke, J. Augustin, F. Feichtner, A.-C. Waselau, C. Klose, H.J. Maier, P. Wriggers, A. Meyer-Lindenberg | Comparison of degradation behaviour and osseointegration of the two magnesium scaffolds, LAE442 and La2, in vivo | 2019 | Excluded; irrelevant comparators |
| 12 | Nan Zhang, Weidan Wang, Xiuzhi Zhang, Krishna. C. Nune, Ying Zhao, Na Liu, R.D.K. Misra, Ke Yang, Lili Tan, Jinglong Yan | The effect of different coatings on bone response and degradation behavior of porous magnesium-strontium devices in segmental defect regeneration | 2020 | Excluded; irrelevant comparators |
| 13 | Olga Charyeva, Ulrich Thormann, Katrin S. Lips, Lydia Heimann, Ursula Sommer, Gabor Szalay, Volker Alt, Norbert Hort, Reinhard Schnettler, Michael Rauschmann, Sven Schmidt | Histological Comparison of New Biodegradable Magnesium Based Implants for Maxillofacial Applications | 2015 | Excluded; irrelevant comparators |
| 14 | Qiang Zhang, Xiao Lin, Zhengrong Qi, Lili Tan, Ke Yang, Zhuangqi Hu, Yan Wang | Magnesium Alloy for Repair of Lateral Tibial Plateau Defect in Minipig Model | 2013 | Excluded; irrelevant comparators |
| 15 | Qing Du, Daqing Wei, Su Cheng, Yaming Wang, Baoqiang Li, Dechang Jia, Yu Zhou | Rapid Structural Evolution and Bone Inducing Mechanism of the Multilayer Coating with Silicon-doped Hydroxyapatite Crystals on the Microwave Water Steaming-Hydrothermally treated Titania Coating | 2020 | Excluded; irrelevant comparators |
| 16 | Shanning Zhang, Xirao Sun, Chunyu Kang, Man Yang, Yuan Zhao, Chengyue Wang | Study on repairing canine mandibular defect with porous Mg–Sr alloy combined with Mg–Sr alloy membrane | 2020 | Excluded; irrelevant comparators |
| 17 | T. Imwinkelried, S. Beck, B. Schaller | Pre-clinical testing of human size magnesium implants in miniature pigs: Implant degradation and bone fracture healing at multiple implantation sites | 2019 | Excluded; irrelevant comparators |
| 18 | Ulrich Thormann, Volker Alt, Lydia Heimann, Cyrille Gasquere, Christian Heiss, Gabor Szalay, Jörg Franke, Reinhard Schnettler, Katrin Susanne Lips | The Biocompatibility of Degradable Magnesium Interference Screws: An Experimental Study with Sheep | 2015 | Excluded; irrelevant comparators |
| 19 | W. Wang, K.C. Nune, L. Tan, N. Zhang, J. Dong, J. Yan, R.D.K.Misra, K. Yang | Bone regeneration of hollow tubular magnesium'strontium scaffolds in critical-size segmental defects: Effect of surface coatings | 2019 | Excluded; irrelevant comparators |
| 20 | Weilin Yu, Huakun Zhao, Zhenyu Ding, Zhiwang Zhang, Benben Sun, Ji Shen, Shanshan Chen, Bingchun Zhang, Ke Yang, Meixia Liu, Daoyun Chen, Yaohua He | In vitro and in vivo evaluation of MgF2 coated AZ31 magnesium alloy porous scaffolds for bone regeneration | 2016 | Excluded; irrelevant comparators |
| 21 | Xingmin Guan, Meiping Xiong, Feiyue Zeng, Bin Xu, Lingdi Yang, Han Guo, Jialin Niu, Jian Zhang, Chenxin Chen, Jia Pei, Hua Huang, Guangyin Yuan | Enhancement of Osteogenesis and Biodegradation Control by Brushite Coating on Mg−Nd−Zn−Zr Alloy for Mandibular Bone Repair | 2014 | Excluded; irrelevant comparators |
| 22 | Y.F. Wu, Y.M. Wang, Y.B. Jing, J.P. Zhuang, J.L.Yan, Z.K. Shao, M.S. Jin, C.J. Wu, Y. Zhou | In vivo study of microarc oxidation coated biodegradable magnesium plate to heal bone fracture defect of 3 mm width | 2017 | Excluded; irrelevant comparators |
| 23 | Yu-Kyoung Kim, Kwang-Bok Lee, Seo-Young Kim, Yong-Seok Jang, Jin Hyeok Kim, Min-Ho Lee | Improvement of osteogenesis by a uniform PCL coating on a magnesium screw for biodegradable applications | 2018 | Excluded; irrelevant comparators |
| 24 | Zheng Xi, Yunfeng Wu, Shouyang Xiang, Chu Sun, Yongxuan Wang, Haiming Yu, Yu Fu, Xintao Wang, Jinglong Yan, Dewei Zhao, Yaming Wang, Nan Zhang | Corrosion Resistance and Biocompatibility Assessment of a Biodegradable Hydrothermal-Coated Mg−Zn−Ca Alloy: An in Vitro and in Vivo Study | 2020 | Excluded; irrelevant comparators |
| 25 | Gururaj Parande, Vyasaraj Manakari, Somasundaram Prasadh, Deep Chauhan, Sarthak Rahate, Raymond Wong, Manoj Gupta | Strength retention, corrosion control and biocompatibility of Mg–Zn–Si/HA nanocomposites | 2019 | Excluded; irrelevant interventions |
| 26 | Hoi Man Wong, Shuilin Wu, Paul K. Chu, Shuk Han Cheng, Keith D.K. Luk, Kenneth M.C. Cheung, Kelvin W.K. Yeung | Low-modulus Mg/PCL hybrid bone substitute for osteoporotic fracture fixation | 2013 | Excluded; irrelevant interventions |
| 27 | Hyung-Sun Youn, M. Anirban Jyoti, Kyung-A Kwak, Hyung-Seok Seo, Byong-Taek Lee, Ho-Yeon Song | Enhanced osteoconduction and angiogenesis of a three dimensional continuously porous Al2O3 implant | 2011 | Excluded; irrelevant interventions |
| 28 | Jun Zhang, Armin Seyfried | Empirical characteristics of different types of pedestrian streams | 2013 | Excluded; irrelevant interventions |
| 29 | Maria Giovanna Ciliberti, Marzia Albenzio, Giovanni Annicchiarico, Agostino Sevi, Antonio Muscio, Mariangela Caroprese | Alterations in sheep peripheral blood mononuclear cell proliferation and cytokine release by polyunsaturated fatty acid supplementation in the diet under high ambient temperature | 2014 | Excluded; irrelevant interventions |
| 30 | Narumi Oshibe, Eriko Marukawa, Tetsuya Yoda, Hiroyuki Harada | Degradation and interaction with bone of magnesium alloy WE43 implants: A long-term follow-up in vivo rat tibia study | 2019 | Excluded; irrelevant interventions |
| 31 | Nasim Golafshan, Morteza Alehosseini, Tahmineh Ahmadi, Ardeshir Talebi, Mohammadhossein Fathi, Mahshid Kharaziha, Gorka Orive, Miguel Castilho, Alireza Dolatshahi-Pirouz | Combinatorial Fluorapatite bioceramic substituted with strontium, magnesium and silicon ions for mending bone defects | 2020 | Excluded; irrelevant interventions |
| 32 | Sujan Krishna Samanta, K. Bavya Devi, Piyali Das, Prasenjit Mukherjee, Abhijit Chanda, Mangal Roy, Samit Kumar Nandi | Metallic ion doped tri-calcium phosphate ceramics: Effect of dynamic loading on in vivo bone regeneration | 2019 | Excluded; irrelevant interventions |
| 33 | Susmita Bose, Sahar Vahabzadeh, Dishary Banerjee, Dongxu Ke | Enhanced bone remodeling on human osteoblast-osteoclast co-culture system using doped hydroxyapatite plasma coatings for musculoskeletal applications | 2019 | Excluded; irrelevant interventions |
| 34 | Abhishek M S, Satish Jaiswal, Anshu Dubey, Debrupa Lahiri, Arup Kumar Das | Biocompatibility and Biodegradability evaluation of Mg-Based Intramedullary Bone Implants in Avian Model | 2020 | Excluded; not using conversion coatings |
| 35 | Arnab Mahato, Munmun De, Promita Bhattacharjee, Vinod Kumar, Prasenjit Mukherjee, Gajendra Singh, Biswanath Kundu, Vamsi K. Balla, Samit Kumar Nandi | Role of calcium phosphate and bioactive glass coating on in vivo bone healing of new Mg–Zn–Ca implant | 2021 | Excluded; not using conversion coatings |
| 36 | Le Hoang Nam Dang, Yu Kyoung Kim, Seo Young Kim, Kuk Jin Lim, Ken Bode, Min Ho Lee, Kwang Bok Lee | Radiographic and histologic effects of bone morphogenetic protein-2/hydroxyapatite within bioabsorbable magnesium screws in a rabbit model | 2019 | Excluded; not using conversion coatings |
| 37 | Mareike Lalk, Janin Reifenrath Nina Angrisani, Alexandr Bondarenko, Jan-Marten Seitz, Peter P. Mueller, Andrea Meyer-Lindenberg | Fluoride and calcium-phosphate coated sponges of the magnesium alloy AX30 as bone grafts: a comparative study in rabbits | 2013 | Excluded; not using conversion coatings |
| 38 | Muhammad Badar, Heinrich Lünsdorf, Florian Evertz, Muhammad Imran Rahim, Birgit Glasmacher, Hansjörg Hauser, Peter P. Mueller | Formation of an organic coat and release of corrosion microparticles from met‐ allic magnesium implants | 2013 | Excluded; not using conversion coatings |
| 39 | Yu Guo, Yanjun Yu, Liping Han, Shanshan Ma, Jinghui Zhao, Huimin Chen, Zukun Yang, Feimin Zhang, Yang Xia, Yanmin Zhou | Biocompatibility and osteogenic activity of guided bone regeneration membrane based on chitosan-coated magnesium alloy | 2019 | Excluded; not using conversion coatings |
| 40 | Zhe Wang, Xinyuan Wang, Yuan Tian, Jia Pei, Jian Zhang, Chang Jiang, Junming Huang, Zhiying Pang, Yuanwu Cao, Xiuhui Wang, Senbo An, Xiao Wang, Hua Huang, Guangyin Yuan, Zuoqin Yan | Degradation and osteogenic induction of a SrHPO4-coated Mg–Nd–Zn–Zr alloy intramedullary nail in a rat femoral shaft fracture model | 2020 | Excluded; not using conversion coatings |
| 41 | Zhengjie Lin, Shuilin Wu, Xuanyong Liu, Shi Qian, Paul K. Chu, Yufeng Zheng, Kenneth M.C. Cheung, Ying Zhao, Kelvin W.K. Yeung | A surface-engineered multifunctional TiO2 based nano-layer simultaneously elevates the corrosion resistance, osteoconductivity and antimicrobial property of a magnesium alloy | 2019 | Excluded; not using conversion coatings |
| 42 | Abudureheman Bahatibieke, Haiming Qin, Tong Cui, Yan Liu, Zixuan Wang | In vivo and in simulated body fluid degradation behavior and biocompatibility evaluation of anodic oxidation-silane-chitosan-coated Mg-4.0Zn-0.8Sr alloy for bone application | 2020 | Excluded; only using composite coatings |
| 43 | Dan Zhang, Ni Ni, Yun Su, Hongwei Miao, Zhimin Tang, Yongrong Ji, Yuyao Wang, Huiqin Gao, Yahan Ju, Na Sun, Hao Sun, Guangyin Yuan, Yinchuan Wang, Huifang Zhou, Hua Huang, Ping Gu, Xianqun Fan | Targeting Local Osteogenic and Ancillary Cells by Mechanobiologically Optimized Mg Scaffolds for Orbital Bone Reconstruction in Canines | 2020 | Excluded; only using composite coatings |
| 44 | Govindaraj Perumal, Boopalan Ramasamy, A Maya Nandkumar, D Sivaraman, R Selvaraj, Mukesh Doble | Bilayered nanostructure coating on AZ31 magnesium alloy implants for the healing of critical-sized rabbit femoral segmental bone defects | 2020 | Excluded; only using composite coatings |
| 45 | Jianan Li, Pei Han, Weiping Ji, Yang Song, Shaoxiang Zhang, Ying Chen, Changli Zhao, Fan Zhang, Xiaonong Zhang, Yao Jiang | The in vitro indirect cytotoxicity test and in vivo interface bioactivity evaluation of biodegradable FHA coated Mg–Zn alloys | 2011 | Excluded; only using composite coatings |
| 46 | Peng Liu, Yuqiang Man, Yusong Bao | Bioactive Porous Biocomposites Coated Magnesium Alloy Implant for Bone Rejuvenation Using a Fracture in Rat Model | 2021 | Excluded; only using composite coatings |
| 47 | Shuang Wu, Yong-Seok Jang, Yu-Kyoung Kim, Seo-Young Kim, Seung-O Ko, Min-Ho Lee | Surface Modification of Pure Magnesium Mesh for Guided Bone Regeneration: In Vivo Evaluation of Rat Calvarial Defect | 2019 | Excluded; only using composite coatings |
| 48 | Shun-Yi Jian, Salim Levent Aktug, Hsuan-Ti Huang, Cheng-Jung Ho, Sung-Yen Lin, Chung-Hwan Chen, Min-Wen Wang, Chun-Chieh Tseng | The Potential of Calcium/Phosphate Containing MAO Implanted in Bone Tissue Regeneration and Biological Characteristics | 2021 | Excluded; only using composite coatings |
| 49 | Soo-Hwan Byun, Ho-Kyung Lim, Sung-Mi Lee, Hyoun-Ee Kim, Soung-Min Kim, Jong-Ho Lee | Biodegradable Magnesium Alloy (ZK60) with a Poly(l-lactic)-Acid Polymer Coating for Maxillofacial Surgery | 2020 | Excluded; only using composite coatings |
| 50 | Soo-Hwan Byun, Ho-Kyung Lim, Soung-Min Kim, Sung-Mi Lee, Hyoun-Ee Kim, Jong-Ho Lee | The Bioresorption and Guided Bone Regeneration of Absorbable Hydroxyapatite-Coated Magnesium Mesh | 2017 | Excluded; only using composite coatings |
| 51 | Y.J. Liu, Z.Y. Yang, L.L. Tan, H. Li, Y.Z. Zhang | An animal experimental study of porous magnesium scaffold degradation and osteogenesis | 2014 | Excluded; only using composite coatings |
| 52 | Yu-Kyoung Kim, Seo-Young Kim, Se Hwan Lee, Min Ho Lee, Kwang-Bok Lee | Stabilized loading of hyaluronic acid-containing hydrogels into magnesium-based cannulated screw | 2019 | Excluded; only using composite coatings |
| 53 | Conor F. Dunne, Galit Katarivas Levy, Orly Hakimi, Eli Aghion, Barry Twomey, Kenneth T. Stanton | Corrosion behaviour of biodegradable magnesium alloys with hydroxyapatite coatings | 2016 | Excluded; without in vivo animal models |
| 54 | E.C. Meng, S.K. Guan, H.X. Wang, L.G. Wang, S.J. Zhu, J.H. Hu, C.X. Ren, J.H. Gao, Y.S. Feng | Effect of electrodeposition modes on surface characteristics and corrosion properties of fluorine-doped hydroxyapatite coatings on Mg–Zn–Ca alloy | 2010 | Excluded; without in vivo animal models |
| 55 | Huawei Yang, Kada Xia, Taolei Wang, Junchao Niu, Yiming Song, Zuquan Xiong, Kui Zheng, Shiqing Wei, Wei Lu | Growth, in vitro biodegradation and cytocompatibility properties of nanohydroxyapatite coatings on biodegradable magnesium alloys | 2016 | Excluded; without in vivo animal models |
| 56 | J.G. Acheson, S. McKillop, P. Lemoine, A.R. Boyd, B.J. Meenan | Control of magnesium alloy corrosion by bioactive calcium phosphate coating: Implications for resorbable orthopaedic implants | 2019 | Excluded; without in vivo animal models |
| 57 | M. Saremi, Sh. Mohajernia, S. Hejazi | Controlling the degradation rate of AZ31 Magnesium alloy and purity of nano-hydroxyapatit coating by pulse electrodeposition | 2014 | Excluded; without in vivo animal models |
| 58 | Peng Wan, Xun Qiu, LiLi Tan, XinMin Fan, Ke Yang | The effects of pulse electrodeposition parameters on morphology and formation of dual-layer Si-doped calcium phosphate coating on AZ31 alloy | 2014 | Excluded; without in vivo animal models |
| 59 | Thiago F. da Conceiç~ao, Nico Scharnagl | Fluoride conversion coatings for magnesium and its alloys for the biological environment | 2015 | Excluded; without in vivo animal models |
| 60 | Mehdi Razavi, Mohammadhossein Fathi, Omid Savabi, Daryoosh Vashaee, Lobat Tayebi | Regenerative influence of nanostructured bredigite (Ca7MgSi4O16)/anodic spark coating on biodegradable AZ91 magnesium alloy implants for bone healing | 2015 | Excluded; irrelevant conversion coatings |
| 61 | Patricia Neacsu, Adela Ioana Staras, Stefan Ioan Voicu, Iuliana Ionascu, Teodoru Soare, Seralp Uzun, Vasile Danut Cojocaru, Andreea Madalina Pandele, Sorin Mihai Croitoru, Florin Miculescu, Cosmin Mihai Cotrut, Ioan Dan, Anisoara Cimpean | Characterization and In Vitro and In Vivo Assessment of a Novel Cellulose Acetate-Coated Mg-Based Alloy for Orthopedic Applications | 2017 | Excluded; irrelevant conversion coatings |
| 62 | Lili Tan, Qiang Wang, Xiao Lin, Peng Wan, Guangdao Zhang, Qiang Zhang, Ke Yang | Loss of mechanical properties in vivo and bone–implant interface strength of AZ31B magnesium alloy screws with Si-containing coating | 2013 | Excluded; irrelevant conversion coatings |
| 63 | Huyang Duan, Chuanliang Cao, XiaoleiWang, JunTao, Chen Li, Hongbo Xin, JingYang, Yulin Song, FanrongAi | Magnesium‑alloy rods reinforced bioglass bone cement composite scafolds with cortical bone‑matching mechanical properties and excellent osteoconductivity for load‑bearing bone in vivo regeneration | 2020 | Excluded; irrelevant conversion coatings |
| 64 | Haitao Xu, Tu Hu, Manle Wang, Yuxin Zheng, Hui Qin, Huiliang Cao, Zhiquan An | Degradability and biocompatibility of magnesium MAO: The consistency and contradiction between in-vitro and in-vivo outcomes | 2018 | Included in SR; excluded from NMA: inapppropriate data form |
| 65 | Jin'e Sun, Jingbo Wang, Hongfeng Jiang, Minfang Chen, Yanze Bi, Debao Liu | In vivo comparative property study of the bioactivity of coated Mg–3Zn–0.8Zr alloy | 2013 | Included in SR; excluded from NMA: inapppropriate data form |
| 66 | Mehdi Razavi, Mohammadhossein Fathi, Omid Savabi, Daryoosh Vashaee, Lobat Tayebi | In vivo assessments of bioabsorbable AZ91 magnesium implants coated with nanostructured fluoridated hydroxyapatite by MAO/EPD technique for biomedical applications | 2014 | Included in SR; excluded from NMA: inapppropriate data form |
| 67 | Mehdi Razavi, Mohammadhossein Fathi, Omid Savabi, Daryoosh Vashaee, Lobat Tayebi | In vivo study of nanostructured akermanite/PEO coating on biodegradable magnesium alloy for biomedical applications | 2014 | Included in SR; excluded from NMA: inapppropriate data form |
| 68 | Mehdi Razavi, Mohammadhossein Fathi, Omid Savabi, Daryoosh Vashaee, Lobat Tayebi | In vivo biocompatibility of Mg implants surface modified by nanostructured merwinite/PEO | 2015 | Included in SR; excluded from NMA: inapppropriate data form |
| 69 | Mehdi Razavi, Mohammadhossein Fathi, Omid Savabi, Seyed Mohammad Razavi, Fariba Heidari, Maziar Manshaei, Daryoosh Vashaee, Lobat Tayebi | In vivo study of nanostructured diopside (CaMgSi2O6) coating on magnesium alloy as biodegradable orthopedic implants | 2014 | Included in SR; excluded from NMA: inapppropriate data form |
| 70 | Mi Hyun Song, Won Joon Yoo, Tae-Joon Cho, Yong Koo Park, Wang-Jae Lee, In Ho Choi | In Vivo Response of Growth Plate to Biodegradable Mg-Ca-Zn Alloys Depending on the Surface Modification | 2019 | Included in SR; excluded from NMA: inapppropriate data form |
| 71 | Yixuan Li, Sheng Zhao, Sirong Li, Yuxiang Ge, Rongliang Wang, Liming Zheng, Jiankun Xu, Minghui Sun, Qing Jiang, Yifeng Zhang, Hui Wei | Surface Engineering of Biodegradable Magnesium Alloys for Enhanced Orthopedic Implants | 2019 | Included in SR; excluded from NMA: inapppropriate data form |
| 72 | Yunfeng Wu, Yaming Wang, Dewei Zhao, Nan Zhang, Hongyu Li, Junlei Li, Yongxuan Wang, Ying Zhao, Jinglong Yan, Yu Zhou | In vivo study of microarc oxidation coated Mg alloy as a substitute for bone defect repairing: degradation behavior, mechanical properties, and bone response | 2019 | Included in SR; excluded from NMA: inapppropriate data form |
| 73 | Jinpeng Zhuang, Yongbin Jing, Yaming Wang, Jinghuai Zhang, Huanxin Xie, Jinglong Yan | Degraded and osteogenic properties of coated magnesium alloy AZ31; an experimental study | 2016 | Included in SR; excluded from NMA: inappropriate outcome assessment |
| 74 | Junjie Han, Peng Wan, Yu Sun, Zongyuan Liu, Xinmin Fan, Lili Tan, Ke Yang | Fabrication and Evaluation of a Bioactive Sr–Ca–P Contained Micro-arc Oxidation Coating on Magnesium Strontium Alloy for Bone Repair Application | 2015 | Included in SR; excluded from NMA: inappropriate outcome assessment |
| 75 | Wei Liu, Tingting Li, Chao Yang, Dong Wang, Guo He, Mengqi Cheng, Qiaojie Wang, Xianlong Zhang | Lithium-Incorporated Nanoporous Coating Formed by Micro Arc Oxidation (MAO) on Magnesium Alloy with Improved Corrosion Resistance, Angiogenesis and Osseointegration | 2019 | Included in SR; excluded from NMA: inappropriate outcome assessment |
| 76 | Wei Peng, Jun-Xiu Chen, Xian-Feng Shan, Yi-Chuan Wang, Fan He, Xue-Jin Wang, Li-Li Tan, Ke Yang | Mg-based absorbable membrane for guided bone regeneration(GBR): a pilot study | 2019 | Included in SR; excluded from NMA: inappropriate outcome assessment |
| 77 | Xing Xiao, Haiying Yu, Qingsan Zhu, Guangyu Li, Yang Qu, Rui Gu | In Vivo Corrosion Resistance of Ca-P Coating on AZ60 Magnesium Alloy | 2013 | Included in SR; excluded from NMA: inappropriate outcome assessment |
| 78 | Yevheniia Husak, Oleksandr Solodovnyk, Anna Yanovska, Yevhenii Kozik, Iryna Liubchak, Viktoriia Ivchenko, Oleg Mishchenko, Yevhen Zinchenko, Vladimir Kuznetsov, Maksym Pogorielov | Degradation and In Vivo Response of Hydroxyapatite-Coated Mg Alloy | 2018 | Included in SR; excluded from NMA: inappropriate outcome assessment |
| 79 | Yu-Kyoung Kim, Young-Seok Jang, Seo-Young Kim, Min-Ho Lee | Functions achieved by the hyaluronic acid derivatives coating and hydroxide film on bio-absorbed Mg | 2018 | Included in SR; excluded from NMA: inappropriate outcome assessment |
| 80 | Jialin Niu, Guangyin Yuan, Yi Liao, Lin Mao, Jian Zhang, Yongping Wang, Feng Huang, Yao Jiang, Yaohua He, Wenjiang Ding | Enhanced biocorrosion resistance and biocompatibility of degradable Mg–Nd–Zn–Zr alloy by brushite coating | 2013 | Included in SR; excluded from NMA: without quantitative analysis data |
| 81 | Junjie Han, Peng Wan, Ye Ge, Xinmin Fan, Lili Tan, Jianjun Li, Ke Yang | Tailoring the Degradation and Biological Response of a Magnesium-Strontium Alloy for Potential Bone Substitutes Application | 2015 | Included in SR; excluded from NMA: without quantitative analysis data |
| 82 | JX Yang, FZ Cui, I-S Lee, Y Zhang, QS Yin, H Xia, SX Yang | In vivo biocompatibility and degradation behavior of Mg alloy coated by calcium phosphate in a rabbit model | 2011 | Included in SR; excluded from NMA: without quantitative analysis data |
| 83 | Qiang Wang, Lili Tan, Wenli Xu, Bingchun Zhang, Ke Yang | Dynamic behaviors of a Ca–P coated AZ31B magnesium alloy during in vitro and in vivo degradations | 2011 | Included in SR; excluded from NMA: without quantitative analysis data |
| 84 | Wei Sun, Guangdao Zhang, Lili Tan, Ke Yang, Hongjun Ai | The fluoride coated AZ31B magnesium alloy improves corrosion resistance and stimulates bone formation in rabbit model | 2016 | Included in SR; excluded from NMA: without quantitative analysis data |
| 85 | Xiangdong Kong, Lei Wang, Guoyuan Li, Xinhua Qu, Jialin Niu, Tingting Tang, Kerong Dai, Guangyin Yuan, Yongqiang Hao | Mg-based bone implants show promising osteoinductivity andcontrollable degradation: A long-term study in a goat femoral condyle fracture model | 2017 | Included in SR; excluded from NMA: without quantitative analysis data |
| 86 | Benoit Schaller, Nikola Saulacic, Stefan Beck, Thomas Imwinkelried, Edwin Wei Yang Liu, Ken Nakahara, Willy Hofstetter, Tateyuki Iizuka | Osteosynthesis of partial rib osteotomy in a miniature pig model using human standard-sized magnesium plate/screw systems: effect of cyclic deformation on implant integrity and bone healing | 2017 | Included in NMA |
| 87 | Benoit Schaller, Nikola Saulacic, Thomas Imwinkelried, Stefan Beck, Edwin Wei Yang Liu, Jan Gralla, Ken Nakahara, Willy Hofstetter, Tateyuki Iizuka | In vivo degradation of magnesium plate/screw osteosynthesis implant systems: Soft and hard tissue response in a calvarial model in miniature pigs | 2016 | Included in NMA |
| 88 | C Iglesias, O G Bodelón, R Montoya, C Clemente, M C Garcia-Alonso, J C Rubio, M L Escudero | Fracture bone healing and biodegradation of AZ31 implant in rats | 2015 | Included in NMA |
| 89 | Chuan-yi Bai, Jian-wu Li, Wan-bao Ta, Bo Li, Yong Han | In vivo Study on the Corrosion Behavior of Magnesium Alloy Surface Treated with Micro-arc Oxidation and Hydrothermal Deposition | 2017 | Included in NMA |
| 90 | H. Naujokat, C.B. Ruff, T. Klu¨ter, J.-M. Seitz, Y. Ac¸il, J. Wiltfang | Influence of surface modifications on the degradation of standard-sized magnesium plates and healing of mandibular osteotomies in miniature pigs | 2019 | Included in NMA |
| 91 | Ho-Kyung Lim, Soo-Hwan Byun, Jae-Man Woo, Sae-Mi Kim, Sung-Mi Lee, Bong-Ju Kim, Hyoun-Ee Kim, Jung-Woo Lee, Soung-Min Kim, Jong-Ho Lee | Biocompatibility and Biocorrosion of Hydroxyapatite-Coated Magnesium Plate: Animal Experiment | 2017 | Included in NMA |
| 92 | Ho-Kyung Lim, Soo-Hwan Byun, Jin-Yong Lee, Jung-Woo Lee, Sae-Mi Kim, Sung-Mi Lee, Hyoun-Ee Kim, Jong-Ho Lee | Radiological, histological, and hematological evaluation of hydroxyapatite-coated resorbable magnesium alloy screws placed in rabbit tibia | 2016 | Included in NMA |
| 93 | Hongfeng Jiang, Jingbo Wang, Minfang Chen, Debao Liu | Biological activity evaluation of magnesium fluoride coated Mg-Zn-Zr alloy in vivo | 2017 | Included in NMA |
| 94 | Hongwei Chai, Lei Guo, Xiantao Wang, Xiaoyu Gao, Kui Liu, Yuping Fu, Junlin Guan, Lili Tan, Ke Yang | In vitro and in vivo evaluations on osteogenesis and biodegradability of a β-tricalcium phosphate coated magnesium alloy | 2011 | Included in NMA |
| 95 | Jie Wang, Feng Peng, Xiaolin Wu, Donghui Wang, Ao Zheng, Lingyan Cao, Chunhua Yu, Xuanyong Liu, Xinquan Jiang | Biocompatibility and bone regeneration of PEO/Mg-Al LDH-coated pure Mg: an in vitro and in vivo study | 2020 | Included in NMA |
| 96 | Mehdi Razavi, Mohammadhossein Fathi, Omid Savabi, Lobat Tayebi, Daryoosh Vashaee | Biodegradable Magnesium Bone Implants Coated with a Novel Bioceramic Nanocomposite | 2020 | Included in NMA |
| 97 | Mike Barbeck, Lennart Kühnel, Frank Witte, Jens Pissarek, Clarissa Precht, Xin Xiong, Rumen Krastev, Nils Wegner, Frank Walther, Ole Jung | Degradation, Bone Regeneration and Tissue Response of an Innovative Volume Stable Magnesium-Supported GBR/GTR Barrier Membrane | 2020 | Included in NMA |
| 98 | Montserrat Rabago Smith, Patrick Atkinson, De´ sire´ e White, Tyler Piersma, Gloria Gutierrez, Gianny Rossini, Sapna Desai, Stephen Wellinghoff, Hui Yu, Xingguo Cheng | Design and assessment of a wrapped cylindrical Ca-P AZ31 Mg alloy for critical-size ulna defect repair | 2011 | Included in NMA |
| 99 | Nan Zhang, Dewei Zhao, Na Liu, Yunfeng Wu, Jiahui Yang, Yuefei Wang, Huanxin Xie, Ye Ji, Changlong Zhou, Jinpeng Zhuang, Yaming Wang, Jinglong Yan | Assessment of the degradation rates and effectiveness of different coated Mg-Zn-Ca alloy scaffolds for in vivo repair of critical-size bone defects | 2018 | Included in NMA |
| 100 | O G Bodelón, CIglesias, J Garrido, CClemente, M C Garcia-Alonso, M L Escudero | Analysis of metallic traces from the biodegradation of endomedullary AZ31 alloy temporary implants in rat organs after long implantation times | 2015 | Included in NMA |
| 101 | S.F. Fischerauer, T. Kraus, X. Wu, S. Tangl, E. Sorantin, A.C. Hänzi, J.F. Löffler, P.J. Uggowitzer, A.M. Weinberg | In vivo degradation performance of micro-arc-oxidized magnesium implants: A micro-CT study in rats | 2012 | Included in NMA |
| 102 | Sae-Mi Kim, Ji-Hoon Jo, Sung-Mi Lee, Min-Ho Kang, Hyoun-Ee Kim, Yuri Estrin, Jong-Ho Lee, Jung-Woo Lee, Young-Hag Koh | Hydroxyapatite-coated magnesium implants with improved in vitro and in vivo biocorrosion, biocompatibility, and bone response | 2013 | Included in NMA |
| 103 | Shuang Wu, Yong-Seok Jang, Min-Ho Lee | Enhancement of Bone Regeneration on Calcium-Phosphate-Coated Magnesium Mesh: Using the Rat Calvarial Model | 2021 | Included in NMA |
| 104 | Wei Wang, Gaozhi Jia, Qing Wang, Hua Huang, Xiaolin Li, Hui Zeng, Wenjiang Ding, Frank Witte, Changqing Zhang, Weitao Jia, Guangyin Yuan | The in vitro and in vivo biological effects and osteogenic activity of novel biodegradable porous Mg alloy scaffolds | 2020 | Included in NMA |
| 105 | Xiao Lin, Lili Tan, Qiang Wang, Guangdao Zhang, Bingchun Zhang, Ke Yang | In vivo degradation and tissue compatibility of ZK60 magnesium alloy with micro-arc oxidation coating in a transcortical model | 2013 | Included in NMA |
| 106 | Zhen Li, ShizhaoSun, Minfang Chen, Bradley Dean Fahlman, Debao Liu, Hongwei Bi | In vitro and in vivo corrosion, mechanical properties and biocompatibility evaluation of MgF2-coated Mg-Zn-Zr alloy as cancellous screws | 2017 | Included in NMA |
