# Supplementary material for: A Systematic Review and Network Meta-Analysis of Biomedical Mg Alloy and Surface Coatings in Orthopedic Application
Source: Bioinorg Chem Appl. 2022 Mar 31;2022:4529520. doi: 10.1155/2022/4529520 (PMC8991394; doi:10.1155/2022/4529520)
Supplement: Supplementary Materials — Figure S1. Contribution graph of % degradation. Figure S2. Contribution graph of new bone formation. Table S1. Searching strategy and result on the PubMed. Table S2. Searching strategy and result on Science Direct. Table S3. Searching strategy and result on Web of Science. Table S4. Included and excluded studies after first screening. [file 4529520.f1.zip › 4529520.f1/(Supplementary materials) Tables S1-3.doc]

| **Search Subject** | **Strategy** | **Result** |
| --- | --- | --- |
| **#1 Type of the study** | **(((((((((bone defect) OR (bone healing)) OR (((((((((((("Fixation, Fracture") OR ("Fixations, Fracture")) OR ("Fracture Fixations")) OR ("Skeletal Fixation")) OR ("Fixation, Skeletal")) OR ("Fixations, Skeletal")) OR ("Skeletal Fixations")) OR ("Fracture Reduction")) OR ("Fracture Reductions")) OR ("Reduction, Fracture")) OR ("Reductions, Fracture")) OR ("Fracture Fixation"[Mesh]))) OR (((((((("Bone Nail") OR ("Nail, Bone")) OR ("Nails, Bone")) OR ("Bone Pins")) OR ("Bone Pin")) OR ("Pin, Bone")) OR ("Pins, Bone")) OR ("Bone Nails"[Mesh]))) OR ((((((((((((((("Broken Bones") OR ("Bone, Broken")) OR ("Bones, Broken")) OR ("Broken Bone")) OR ("Bone Fractures")) OR ("Bone Fracture")) OR ("Fracture, Bone")) OR ("Spiral Fractures")) OR ("Fracture, Spiral")) OR ("Fractures, Spiral")) OR ("Spiral Fracture")) OR ("Torsion Fractures")) OR ("Fracture, Torsion")) OR ("Fractures, Torsion")) OR ("Torsion Fracture"))) OR ((("Bone Screw") OR ("Screw, Bone")) OR ("Screws, Bone"))) OR ((("Bone Plate") OR ("Plate, Bone")) OR ("Plates, Bone"))) OR ("Bone Plates"[Mesh])) OR ("Bone Screws"[Mesh])** | **117208** |
| **#2 intervention** | **(((((((((((((((((Ca-P[Title/Abstract]) OR (Calcium phosphate[Title/Abstract])) OR (HAP[Title/Abstract])) OR (HA[Title/Abstract])) OR (hydroxyapatite[Title/Abstract])) OR (CaHPO4[Title/Abstract])) OR (Brushite[Title/Abstract])) OR (MAO[Title/Abstract])) OR (micro-arc oxidation[Title/Abstract])) OR (microarc oxidation[Title/Abstract])) OR (microarc oxidized[Title/Abstract])) OR (PEO[Title/Abstract])) OR (MgF2[Title/Abstract])) OR (fluoride[Title/Abstract])) OR (phytic acid[Title/Abstract])) OR (PA[Title/Abstract]) AND ((Magnesium Alloy[Title/Abstract]) OR (Mg[Title/Abstract])** | **2891** |
| **#3 years** | **in the last 10 years** |  |
| **#4 object of study** | Search filter for animal studies |  |
| **#5 Combination of all search key words** |  | **164** |

Table S1. Searching strategy and result on the PubMed

| **Search Subject** | **Strategy** | **Result** |
| --- | --- | --- |
| **#1 Find articles with these terms** | ("Bone Screws") OR ("Bone Plates") OR ("Fractures Bone") OR ("Fracture Fixation") OR ("Bone Nails") OR (bone healing) OR (bone defect) | 180985 |
| **#2 years** | 2011-2021 |  |
| **#3 Title, abstract or author-specified keywords** | ((Ca-P) OR (Calcium phosphate) OR (HAP) OR (hydroxyapatite) OR (CaHPO4) OR (Brushite)) AND ((Magnesium Alloy) OR (Mg)) AND (vivo) | 273 |
| **#4 Combination of all search key words** |  | 110 |
| **Search Subject** | **Strategy** | **Result** |
| **#1 Find articles with these terms** | ("Bone Screws") OR ("Bone Plates") OR ("Fractures Bone") OR ("Fracture Fixation”) OR ("Bone Nails") OR (bone healing) OR (bone defect) | 180985 |
| **#2 years** | 2011-2021 |  |
| **#3 Title, abstract or author-specified keywords** | ((MAO) OR (micro-arc oxidation) OR (microarc oxidation) OR (microarc oxidized) OR (PEO)) AND ((Magnesium Alloy) OR (Mg)) AND (vivo) | 62 |
| **#4 Combination of all search key words** |  | 28 |
| **Search Subject** | **Strategy** | **Result** |
| **#1 Find articles with these terms** | ("Bone Screws") OR ("Bone Plates") OR ("Fractures Bone") OR ("Fracture Fixation”) OR ("Bone Nails") OR (bone healing) OR (bone defect) | 180985 |
| **#2 years** | 2011-2021 |  |
| **#3 Title, abstract or author-specified keywords** | ((MgF2) OR (fluoride) OR (phytic acid) OR (PA)) AND ((Magnesium Alloy) OR (Mg)) AND (vivo) | 105 |
| **#4 Combination of all search key words** |  | 13 |

Table S2. Searching strategy and result on Science Direct

| **Search Subject** | **Strategy** | **Result** |
| --- | --- | --- |
| **#1 intervention** | (((TS=(Mg)) OR TS=(Mg alloy)) OR TS=(magnesium)) AND ((((((((((((((((TS=(Ca-P)) OR TS=(Calcium phosphate)) OR TS=(HAP)) OR TS=(HA)) OR TS=(hydroxyapatite)) OR TS=(CaHPO4)) OR TS=(Brushite)) OR TS=(MAO)) OR TS=(micro-arc oxidation)) OR TS=(microarc oxidation)) OR TS=(microarc oxidized)) OR TS=(PEO)) OR TS=(MgF2)) OR TS=(fluoride)) OR TS=(phytic acid)) OR TS=(PA)) | 87,335 |
| **#2 object of study** | (TS=(in vivo)) OR TS=(animal experiment) | 2,183,529 |
| **#3 Type of the study** | (((((((TS=(bone fracture)) OR TS=(bone screws)) OR TS=(bone plates)) OR TS=(fracture)) OR TS=(fracture fixation)) OR TS=(bone healing)) OR TS=(bone defect)) OR TS=(bone nails) | 949,809 |
| **#5 Combination of all search key words** | #1, #2 AND #3 | 558 |
| **#5 Final results with the limitation in Publication date** | Search filter for the publication year from 2011 to 2021 | 425 |

Table S3. Searching strategy and result on Web of Science
